# Feasibility of informing syndrome-level empiric antibiotic recommendations using publicly available antibiotic resistance datasets

**DOI:** 10.12688/wellcomeopenres.15477.2

**Published:** 2020-06-24

**Authors:** Quentin J. Leclerc, Nichola R. Naylor, Alexander M. Aiken, Francesc Coll, Gwenan M. Knight

**Affiliations:** 1 Department of Infectious Disease Epidemiology, London School of Hygiene & Tropical Medicine, London, WC1E 7HT, UK; 2 Centre for Mathematical Modelling of Infectious Diseases (CMMID), London School of Hygiene & Tropical Medicine, London, WC1E 7HT, UK; 3 Imperial College Healthcare NHS Trust, London, UK; 4 Department of Infection Biology, London School of Hygiene & Tropical Medicine, London, WC1E 7HT, UK

**Keywords:** Antimicrobial resistance, empiric therapy, guidelines, online tool, data linkage

## Abstract

**Background:** Antibiotics are often prescribed empirically to treat infection syndromes before causative bacteria and their susceptibility to antibiotics are identified. Guidelines on empiric antibiotic prescribing are key to effective treatment of infection syndromes, and need to be informed by likely bacterial aetiology and antibiotic resistance patterns. We aimed to create a clinically-relevant composite index of antibiotic resistance for common infection syndromes to inform recommendations at the national level.

**Methods:** To create our index, we used open-access antimicrobial resistance (AMR) surveillance datasets, including the ECDC Surveillance Atlas, CDDEP ResistanceMap, WHO GLASS and the newly-available Pfizer ATLAS dataset. We integrated these with data on aetiology of common infection syndromes, existing empiric prescribing guidelines, and pricing and availability of antibiotics.

**Results:**  The ATLAS dataset covered many more bacterial species (287) and antibiotics (52) than other datasets (ranges = 8-11 and 16-32 respectively), but had a similar number of samples per country per year. Using these data, we were able to make empiric prescribing recommendations for bloodstream infection, pneumonia and cellulitis/skin abscess in up to 44 countries. There was insufficient data to make national-level recommendations for the other six syndromes investigated. Results are presented in an interactive web app, where users can visualise underlying resistance proportions to first-line empiric antibiotics for infection syndromes and countries of interest.

**Conclusions:** We found that whilst the creation of a composite resistance index for empiric antibiotic therapy was technically feasible, the ATLAS dataset in its current form can only inform on a limited number of infection syndromes. Other open-access AMR surveillance datasets are largely limited to bloodstream infection specimens and cannot directly inform treatment of other syndromes. With improving availability of international AMR data and better understanding of infection aetiology, this approach may prove useful for informing empiric prescribing decisions in settings with limited local AMR surveillance data

## Introduction

Worldwide, most bacterial infections are treated empirically, meaning that antibiotics are prescribed based on clinical judgement prior to the infectious agent and its susceptibilities to antibiotics determined by diagnostic tests ^1^. A point prevalence survey of antibiotic-prescribing in children showed that globally over 75% of antibiotics in neonatal treatment were given empirically ^2^. In low- and middle-income countries, the laboratory capacity that could inform appropriate empiric therapy choices is frequently lacking.

Empiric antibiotic prescribing guidelines contain recommendations on what antibiotics to use for specific infection syndromes. An infection syndrome is a clinical situation where the presence of a specific type of infection (e.g. pneumonia, urinary tract infection) is suspected. Such an infection syndrome might thus be caused by bacteria or a virus or other transmissible pathogens or even might be due to non-infectious inflammatory causes. Here we assume that a decision has already been made that antibiotics are required (i.e. there is a bacterial cause of the syndrome) and do not address the other key diagnostic issue of bacterial or viral agent. The use of prescribing guidelines has been associated with reductions in patient mortality ^3^, particularly among the most critically ill patients ^4^, though benefits vary by patient group and infection ^5^. Guidelines have also been shown to reduce levels of inappropriate prescribing ^6^, which leads to a reduction in the selective pressure for antimicrobial resistance (AMR). Empiric guidelines are important in low-income settings where microbiological confirmation rarely occurs due to infrastructural and resource constraints ^6, 7^.

Guidelines for empiric antibiotic therapies are often set at the national level. For example, in England, Public Health England and the National Institute of Health and Care Excellence (NICE) produce national antimicrobial prescribing guidance ^8^. Creation of such guidelines requires an understanding of both the aetiology (typical causative pathogens) and the prevalence of relevant antibiotic susceptibilities. Each anatomic site of infection (e.g. respiratory tract, urinary tract, skin and soft tissue, gastrointestinal tract) has typical infecting microorganisms. The aetiology of some infection syndromes and associated antibiotic susceptibilities varies by setting, age and even season (as is the case for pneumonia for example ^9^, but some broad generalizations can be made, especially with the broad-spectrum nature of some antibiotic agents.

Though it is recommended that prescribing guidelines should be adapted by healthcare institutions to take into account local patterns of AMR, in practice, this is infrequently performed ^10^. This may be due to a lack of resources to develop appropriate guidelines or a lack of appreciation of the need ^11^ – furthermore, the existence of guidelines is no guarantee that local prescribers will adhere to such recommendations. Providing readily available, easy-to-use, transparently created tools based on open-access international AMR surveillance data may help practitioners in resource-limited settings generate appropriately-tailored local prescribing guidelines.

Whilst antibiotic resistance levels and other clinical criteria form the basis for designing antibiotic prescribing guidelines, in practice, antibiotic use is also constrained by market factors, such as cost and access to antibiotics. This may be particularly true in the case of low- and middle-income countries, which can have limited healthcare budgets and access to medicines. Two antibiotic market factors which can be informed through open-access data are those of antibiotic supplier prices and antibiotic placement on the World Health Organization’s (WHO’s) Essential Medicines List ^12^.

Currently, antibiotic resistance surveillance tools typically present resistance data for individual bacteria-antibiotic (“bug-drug”) combinations ^13^. We use a more clinically-oriented presentation of resistance proportions at the level of infection syndromes, which could be used to inform empiric antibiotic prescribing recommendations. Similar "indices" have been previously proposed ^14^. The Drug Resistance Index was developed to quantify resistance to multiple antibiotics for individual bacterial species ^13^ and communicate to policymakers and non-experts the combined impact resistance has on the antibiotics available for treatment, without directly supporting clinical care. A similar index, but used to assess the population-level appropriateness of empiric treatment regimens for complicated UTI in the Netherlands was also explored by Ciccolini *et al.* ^7^ where the relative frequency of causative agents and frequency of resistance was combined with antibiotic usage data. A study in a Canadian intensive care unit explored the likely efficacy of empiric treatment for three device-associated infections by creating a composite syndrome level resistance ^15^. A “basket” of bacterial agents causing each infection was used similarly to how economists measure the average price of a standard basket of consumer goods weighted by the relative importance of each good ^16^. There are also the weighted incidence syndromic combination antibiograms, which aim to inform empiric prescribing by considering the local weighted incidence of causative pathogens for an infection syndrome ^17^. Thus there are several examples of empiric therapy indices, however, they are either setting and/or syndrome specific, and do not present other potentially important information such as measures of drug access and/or cost information alongside clinical data.

We test the feasibility and robustness of creating a syndrome-level composite resistance index from open-access data sources, including international AMR surveillance datasets, and developed a user-friendly web-based application, the AR.IA App ^18^, that brings together all this information. The app does not aim to be a predictor for likelihood of viral versus bacterial infections, but rather to aid antibiotic prescribing choice where the infectious agent is presumed to be bacterial. This work was undertaken as part of the Wellcome Data Re-use Prize ^19^, motivated by the release of a new open-access dataset (ATLAS) from Pfizer that contained 633,820 bacterial clinical isolates collected from 77 countries over a 14-year period ^19^.

## Methods

This work consists of three main objectives, where specific methods applied:

1. To compare antibiotic resistance proportions calculated using the ATLAS dataset with those estimated from other global AMR surveillance datasets.
2. To integrate data on antibiotic susceptibilities from the ATLAS dataset with the aetiology of infection syndromes to derive a syndrome-level composite resistance index; and combine such data with access to and cost of antibiotics.
3. To develop an interactive web app (AR.IA App) to access the above information and offer empiric therapy recommendations based on available data.

All of the above was conducted in R software ^20^, using the following packages: shiny 1.2.0 ^21^, ggplot2 3.1.0 ^22^, dplyr 0.7.8 ^23^, rworldmap 1.3-1 ^24^, RColorBrewer 1.0-5 ^25^, reshape2 1.4.2 ^26^, DT 0.5 ^27^, magrittr 1.0.1 ^28^, fuzzyjoin 0.1.4 ^29^. These are available at: https://cran.r-project.org/.

### Surveillance data comparison

The ATLAS dataset (available for download at https://amr.theodi.org/programmes/atlas) is an open-access dataset on human AMR surveillance data generated by the commercial pharmaceutical company Pfizer that contains granular antibiotic susceptibility data, including ‘raw’ minimum inhibitory concentration (MIC) data, for 633,820 bacterial clinical isolates collected from 77 countries and spanning 14 years ^30^. This dataset also contains information on the gender and age group (age groups are: 0–2, 3–12, 13–18, 19–64, 65–84 and 85+) of the patients isolates were collected from. It also contains the specimen clinical source, indicating the anatomical site isolates were sampled from (e.g. skin, blood, nose, etc.). The ATLAS dataset was made publicly viewable in 2017, and downloadable in 2018 as part of the Wellcome Data Reuse Prize ^19^. This was an initiative to encourage reuse of AMR data shared by industry and to facilitate the development of common methodological and metadata standards. 

We additionally used the European Centre for Disease Prevention and Control (ECDC) Surveillance Atlas ^31^, ResistanceMap by the Center for Disease Dynamics Economics and Policy (CDDEP) ^32^ and the Global Antimicrobial Resistance Surveillance System (GLASS) database by the World Health Organization (WHO) ^33^. The first holds AMR data collected in European countries whilst the second and third hold global data from national AMR surveillance programmes. ResistanceMap and GLASS both include all of the ECDC dataset, since they use it as a source for their European sepsis data. Data between 2004 and 2017 were considered to match the ATLAS time coverage, but only from 2017 for the GLASS dataset (the only year available for download at the time of our analysis). Missing susceptibility labels (i.e. “resistant”, “intermediate” or “susceptible”) in the ATLAS dataset (443,899/633,820) were assigned from available MIC data for other isolates within the ATLAS dataset. We did not use any external data on breakpoints to derive susceptibility labels for MICs which were not labelled elsewhere within the ATLAS dataset (see *Further Methods* in *Extended Data* for details) ^34^.

We estimated the “agreement” of the ATLAS dataset as the percentage of resistance proportions for all bug-drug combinations with point estimates falling within the corresponding 95% confidence intervals in the ECDC Surveillance Atlas and ResistanceMap databases (see *Further Methods* in *Extended Data* for details) ^34^. Sample sizes (i.e. number of samples per country per year in each dataset, where one sample is one combination of bacteria and antibiotic) were compared using boxplots. We matched susceptibility labels across datasets by assigning all isolates as “resistant” if they were non-susceptible (i.e. “intermediate” or “resistant”).

### Data integration and Mapping

**Figure 1.**
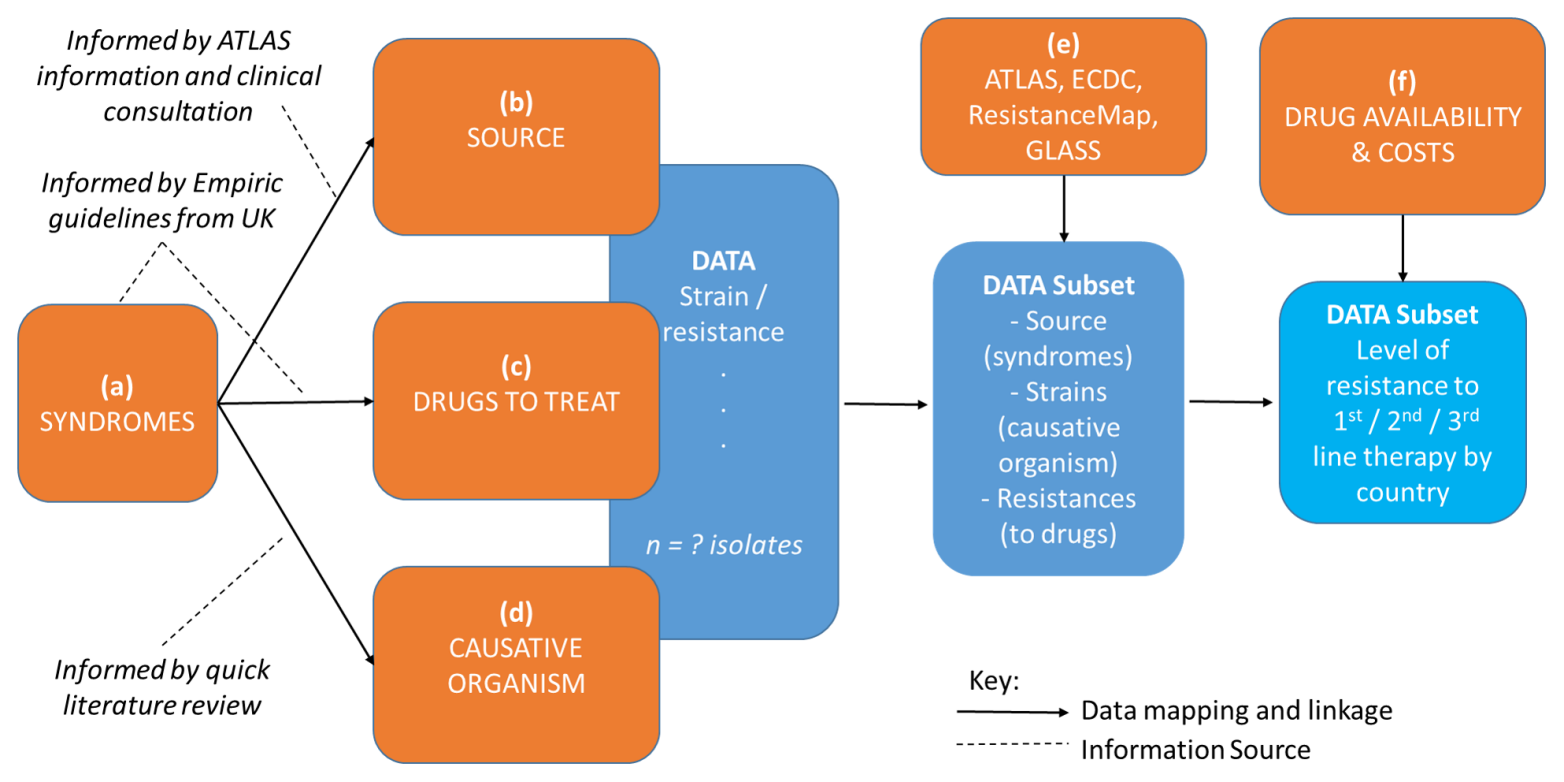
Flowchart of data analysis and inputs for this project. Orange boxes indicate different datasets from which information was extracted. Dark blue boxes represent the initial and subset ATLAS dataset used to inform levels of resistance (light blue box) to standard empiric therapy. Solid arrows joining boxes indicate mapping between data types. Dashed arrows are used to indicate the sources data were extracted from. The direction of the arrows indicate the order data types were extracted and integrated.

Figure 1 shows the steps required to extract and integrate information from sources other than the ATLAS dataset to produce our composite resistance index. We focused on nine infection syndromes. Each infection syndrome was mapped to the corresponding causative bacteria (i.e. aetiology, informed by the scientific literature), antibiotics used to treat them empirically (informed by antibiotic prescribing guidelines and clinical consultation) and related specimen sources (informed by the ATLAS metadata and clinical consultation).

***(a) Common infection syndromes***. We first chose which infection syndromes to focus on. A comprehensive list of infection syndromes was extracted from NICE guideline ^8^. We discarded syndromes predominantly caused by viral and fungal infections and kept the nine most common bacterial ones. Clinically, these syndromes are all identifiable with simple clinical examination and/or basic investigations, and occur worldwide.

***(b) Mapping isolate source to infection syndrome***. We linked isolates in the ATLAS dataset to the infection syndrome they most likely originated from informed by the clinical “source” description in the ATLAS metadata. Due to the diversity of sources, we kept sample types represented by at least 1,000 isolates. We discarded sample types not clearly linked to an infection syndrome (e.g. “wound”), as these samples might not necessarily represent infecting organisms, but rather colonizing bacterial flora.

***(c) Antibiotics used to treat empirically infection syndromes***. We extracted which antibiotics are used to treat empirically each of the nine infection syndromes from the NICE guidelines ^8^ and then took a simplified set as advised by clinical consultation. These empiric therapies represent typical current practice in the UK, though we attempted to make use of agents that were widely available at low cost internationally. For simplicity, we did not incorporate additional patient-level prescribing criteria (such as penicillin allergy status and pregnancy) when choosing these antibiotics, hence the need for clinical consultation alongside the complex NICE guidelines.

***(d) Contributing pathogen distribution: syndrome aetiology***. To establish the distribution of causative pathogens for each infection syndrome, we identified reviews on the global aetiology for different syndromes and, if we could not find any, performed a rapid literature search for recent publications. Rapid, informal reviews were done by three of the authors (NRN, QJL, GMK). Individual syndromes were investigated by each author and searches of the syndrome plus terms like “aetiology” were performed in PubMed and Google in January 2019. Based on the literature found, the percentage of each infection syndrome caused by a bacterial agent were extracted from each study into a prespecified Excel data extraction sheet. One author (GMK) then integrated all of these results, per syndrome, creating a suggested pathogen distribution for each syndrome. Even where non-bacterial (i.e. viral or fungal) pathogens were found to be causative of the syndrome in the literature, only the proportion of the relevant bacterial pathogens were used in this distribution.

***(e) Combining antibiotic susceptibility data from four AMR surveillance datasets***. We extracted the antibiotic susceptibility data from the ATLAS dataset as well as from three more AMR surveillance datasets: ECDC, ResistanceMap and GLASS, to allow the end-user of our AR.IA App to select the underlying antibiotic susceptibility data.

**Figure 2.**
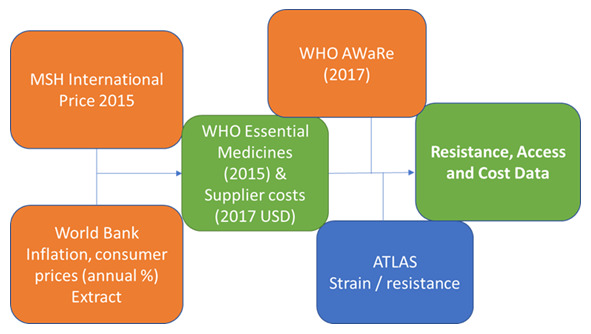
Flowchart of data linkage for further data. Orange boxes indicate additional datasets, blue boxes indicate ATLAS data and green boxes indicate resulting data utilised within this project.

***(f) Combining with drug information datasets***. We extracted data on supplied cost (and cost unit) for antibiotics from the Management Sciences for Health (MSH) International Medical Products Price Guide ^35^ which we inflated to the 2017 level using World Bank inflation data ^36^. We also included whether a recommended drug was on the WHO Essential Medicines List (EML) and on the AWaRE classification system, which builds on the EML to advise on what antibiotics to use for common infections (“access” category), for a small number of infections (“watch” category) and to be considered as last-resort options (“reserve” category) ^37^ ( Figure 2).

**Figure 3.**
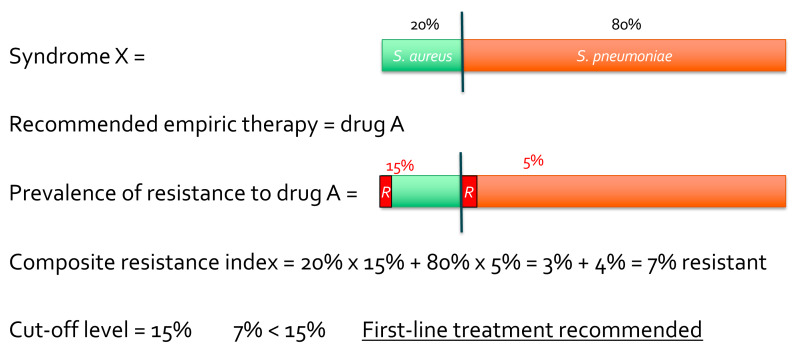
Example of calculation of the composite resistance index. We designed a simple hierarchical decision workflow ( Figure 4) to inform on the appropriateness of using first-line empiric antibiotic therapy by comparing the syndrome-level composite resistance index calculated for each country against the chosen resistance cut-off, defined as the resistance proportion above which to escalate therapy, which is set to 15% by default in the AR.IA App.

***(g) Mapping data to recommendations for therapy***. We multiplied the frequency of each syndrome’s contributing bacteria by their resistance proportion to calculate a composite resistance index for empirically used antibiotics. An example of a made-up syndrome caused by two bacterial species can be seen in Figure 3. In this example, the composite resistance to the first-line antibiotic A is 7%, and since this is less than the default 15% cut-off, first-line treatment A would still be recommended. 

**Figure 4.**
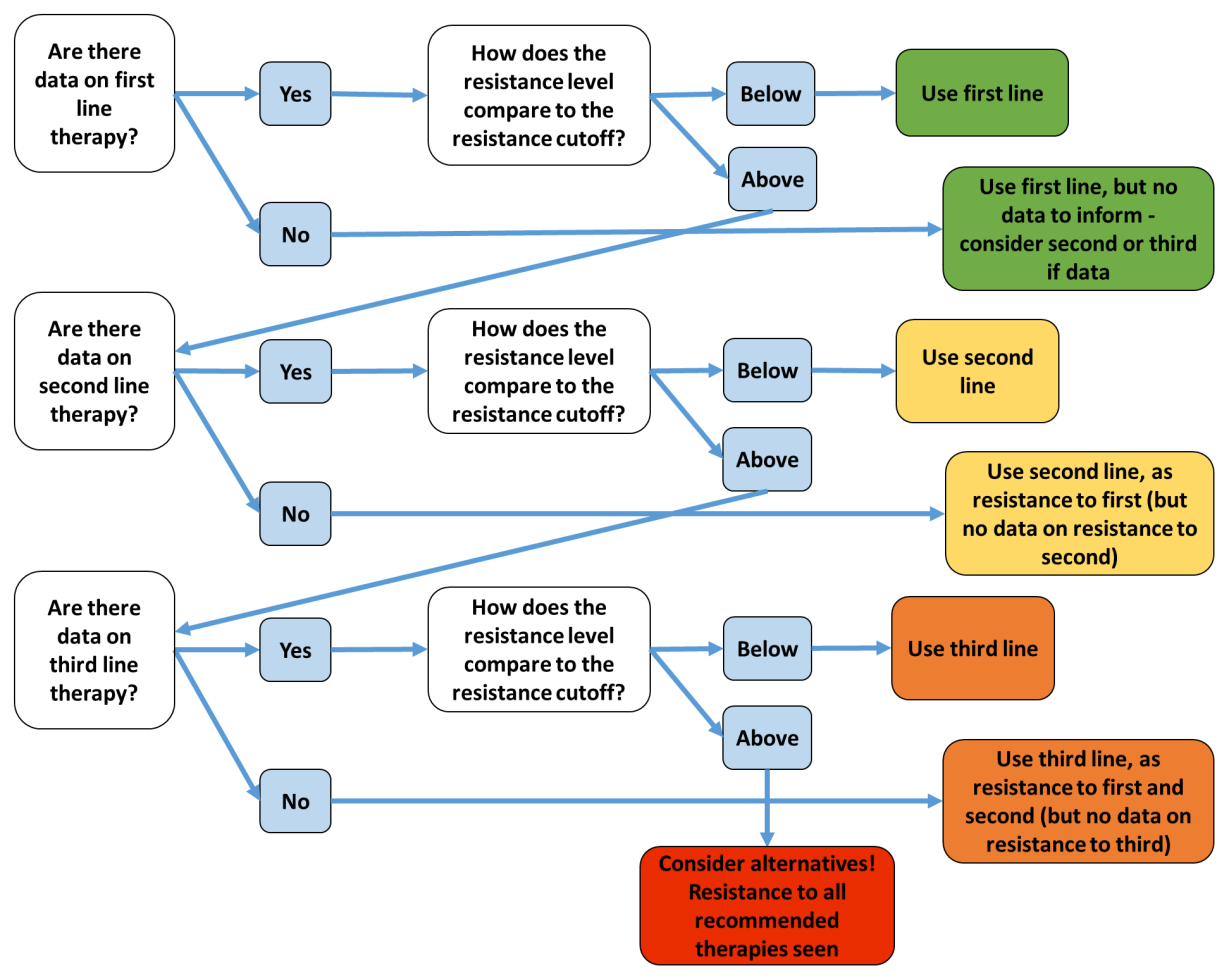
Description of decision-making process for deciding recommendations for treatment. If there is no data to inform therapy, we assume that the bacteria are susceptible and recommend the therapy, with a disclaimer that we have no data to inform. Note here that resistance is at the level of the syndrome. The “...” indicate where the decision making would continue onto third and higher-level therapy options.

Each infection syndrome was assumed to be caused entirely by bacterial species, based on the scope of our work. We noted that not all bacterial species were included in the ATLAS database, nor were all species tested for the antibiotics included in empiric therapies. We thus define the “causative pathogen availability” as the proportion of isolates from available species out of all syndrome-contributing species. For example, if 20% of the syndrome cases are due to bacteria X, 80% due to bacteria Y, but bacteria Y is not in the ATLAS database then the “causative pathogen availability” is 20%. We further looked at susceptibility coverage – not all species were tested for resistance to all drugs. Thus if only 50% of bacteria X had only be tested for resistance to all empiric therapies the coverage would only be 10% in this example. The composite resistance index is then calculated using the resistance proportions of available causative pathogens, assuming missing bacteria to be totally susceptible, which may bias towards using first line therapies. We report the causative pathogen availability, as well as whether fewer than 10 isolates were available in the final recommendation table, but do not set a minimum threshold.

### The AR.IA App creation

The Shiny R package ^21^ was used to build an interactive web app (referred to as ‘the AR.IA App’) that hosts data from all four AMR surveillance datasets, integrated with external information, and the main output data used to recommend what antibiotics are appropriate to treat common infection syndromes in different regions of the world. Key parameters can be edited by the user including the underlying antibiotic susceptibility surveillance dataset used, the syndrome aetiology (proportion due to each included bacterial species) and the resistance cut-off for changing empiric therapy, which is set by default to 15% (see *Usage of the AR.IA App* in *Extended Data* for details) ^38^. The underlying data manipulation and Shiny R code can be found on GitHub.

## Results

### Surveillance data comparison

We used four open-access datasets: ATLAS ^30^, ECDC ^31^, ResistanceMap ^32^ and GLASS ^33^ ( Table 1).

**Table 1.** Descriptors of surveillance datasets.

**Database**	ATLAS	ECDC	ResistanceMap	GLASS
**Created by**	Pfizer	ECDC	CCDEP	WHO
**Year utilised in AR.IA App**	2017	2017	2015	2017
**Types of samples**	All clinical samples	Blood (majority) and cerebrospinal fluid	Blood (majority) and cerebrospinal fluid	Blood, urine, genital and stool
**Geographical area covered**	Global	Europe	Global *	Global *
**Number of countries covered**	77	30	92	69
**Bacterial species covered**	287	8	11	8
**Antibiotics tested**	52	16	20	32
**Sample size**	1,689,267	1,299,941	1,288,325	1,683,765

*with ECDC data for European blood samples

Assigning susceptibility labels from MIC values in the ATLAS dataset reduced the number of isolates with incomplete records by 63% (to 164,918 isolates). The number of isolates per country and year was similar across datasets ( *Extended data*, Supplementary Figures 2 & 3 in *Further Results*) ^39^ . The ATLAS dataset reports many more bacterial species and antibiotics tested than any other dataset ( Table 1), resulting in smaller sample sizes for each country/year/species/antibiotic combination. The “agreement” of the ATLAS dataset by year as compared to the ECDC or ResistanceMap datasets ranged from 5–30% ( *Extended data*, Supplementary Figure 1A in *Further Results*) ^39^. This agreement increases as the ATLAS sample size increases, suggesting that the low agreement is driven in part by small samples sizes, but stays around 25% after sample size exceeds 30,000 ( *Extended data*, Supplementary Figure 1B in *Further Results*), pointing to differences in sampling as the main cause of the low agreement ^39^.

### Data integration and mapping

***(a) Common infection syndromes***. The nine chosen infection syndromes are shown in Table 2. These were chosen as they could be clearly linked to anatomic site or sample types, are common infections worldwide, and are caused by common bacterial species.

**Table 2.** Isolate, syndrome and antibiotic mapping results.

Infection Syndrome	Isolate source in ATLAS	Number of isolates in ATLAS	Total number of isolates in ATLAS	First Line	
				First Drug	Second Drug
Bloodstream infection	CVS: Blood	104,148	104,148	Amoxicillin	Gentamicin
Urinary tract infection	GU: Urine	82,086	84,689	Trimethoprim	-
	GU: Urinary Bladder	2,603			
Pneumonia	Respiratory: Sputum	81,669	122,291	Co-amoxiclav (Hospital- acquired) Amoxicillin (Community- acquired)	- Clarithromycin (Community-acquired)
	Respiratory: Bronchials	25,032			
	Respiratory: Bronchoalveolar lavage	8,335			
	Respiratory: Other	4,412			
	Respiratory: Lungs	2,843			
Cellulitis / skin abscess	INT: Abscess	15,904	33,374	Flucloxacillin	-
	INT: Skin	7,257			
	INT: Skin Ulcer	5,674			
	INT: Cellulitis/Erysipelas	2,501			
	INT: Burn	2,038			
Purulent urethritis / cervicitis	Genital/Urinary (GU)	1,416	2,613	Ceftriaxone	-
	GU: Urethra	1,197			
Upper respiratory tract infection	HEENT: Ears	5,127	15,785	Penicillin V	-
	HEENT: Throat	3,725			
	HEENT: Nose	3,197			
	Respiratory: Sinuses	2,415			
	HEENT: Other	1,321			
Bacterial meningitis	Bodily Fluids: CSF	1,811	1,811	Penicillin	Gentamicin
Septic arthritis	Bodily Fluids: Synovial	1,290	1,290	Oxacillin	-

***(b) Mapping isolate source to infection syndrome***. We could map 366,001/633,820 isolates (58%) from the ATLAS dataset to the syndrome they likely originated from ( Table 2). The major sources excluded were “INT: Wound” (n=96,306 isolates) and “Respiratory: Trachea” (n=19,278) as they could not be linked to a single syndrome and could represent colonizing flora. There was no accompanying clinical information available to help discriminate genuine infecting organisms from colonizers. The isolate “source” information was not sufficient to assign respiratory specimens ( Table 2) to either community or hospital acquired pneumonia, thus we used the same pool of respiratory isolates for both syndromes, but with a different etiological make-up.

***(c) Antibiotics used to treat infection syndromes empirically***. The antibiotics used to treat the nine infection syndromes empirically are shown in Table 2 ( *Extended data*, second line and third line presented in Supplementary Table 1 in *Further Results*) ^39^. Some of the antibiotics recommended for treatment were not tested against in the ATLAS dataset and thus we mapped them to their equivalent tested antibiotic where possible ( *Extended data*, Supplementary Table 2 in *Further Results*) ^39^.

***(d) Contributing pathogen distribution: syndrome aetiology***. Except for bacterial meningitis, we could not find a consensus global aetiology for each infection syndrome. There are liable to be some regional differences in the aetiology of infections and also greater difficulty in obtaining reliable microbiological diagnosis in some parts of the world. We therefore relied on rapid literature reviews to find an approximate breakdown of the top bacterial species commonly isolated from each type of infection (see *Further Results* in *Extended Data* for details) ^39^. However, the AR.IA App allows the bacterial aetiology to be changed by the user. Our syndrome aetiology from the literature included a total of 19 bacterial species. Of these, two were not present in the ATLAS dataset, including " *Streptococcus, viridans* group" and *Neisseria gonorrhoeae*, the latter responsible for the majority of purulent urethritis/cervicitis cases. We therefore excluded the latter syndrome from the AR.IA App.

***(e) Combining antibiotic susceptibility data from four AMR surveillance datasets***. We aggregated antibiotic susceptibility data across the four AMR datasets (ATLAS, ECDC, ResistanceMap and GLASS) by keeping isolates from the most recent year available (2017) for all except ResistanceMap, which had very few data points for 2016 and 2017 (n=46 and n=197, respectively) and so we used 2015 instead (n=684); by standardizing the spelling of antibiotics, species and countries; and by mapping single antibiotics in the ATLAS dataset to their corresponding antibiotic classes (reported in the rest of datasets). When combining the datasets, we average the resistance proportions reported in each dataset for each combination of country, bacteria and antibiotic class. We mapped each isolate source to their relevant infection syndrome as done for the ATLAS dataset. Datasets included isolates from different infection syndromes ( Table 1).

***(f) Combining with drug information datasets***. Approximately 75% of the antibiotics tested in the ATLAS database and used to treat the chosen syndromes were found on the 2015 EML list and over 80% in the AWaRE classification system (see *Further Results* in *Extended Data* for details) ^39^. Only one of these antibiotics (fosfomycin) is classified on the AWaRe “reserve” group. As cost comparisons are difficult across different antibiotics that have different formulations, we allow the AR.IA App user to see exactly which formulation the available costs relate to by presenting the cost in “per specified unit”.

***Summary of data integration and sub-setting***. Following steps (a) - (f) above, we kept isolates in the ATLAS dataset that met the following criteria: were isolated from sources that could be mapped to infection syndromes ( Table 2), belong to the list of bacterial species causing infection syndromes ( Table 3), had assigned susceptibility status (susceptible/resistant) to at least one of the antibiotics used to treat infection syndromes, and were collected in 2017.

**Table 3.** Summary table of the recommendations for therapy. Causative pathogen availability: the average proportion (over all countries) of syndrome causing bacterial species which had susceptibility information available from the dataset. We only include recommendations for syndromes with a causative pathogen availability of at least 0.5. If the composite resistance index is above 15% for an antibiotic, therapy is escalated to the next line.

Syndrome	Mean causative pathogen availability (S.D.)	Recommendation (as displayed in the app)	Number of countries	Therapy	Key driver
**Bloodstream** **infection**	0.70 (0.14)	Use second line, as resistance to first (but no data on resistance to second)	2	Cefuroxime and Gentamicin	*S. aureus* resistance
		Consider alternatives! Resistance to all recommended therapies seen	42		
**Community** **acquired** **pneumonia**	0.83 (0.18)	Use first line, but no data to inform – consider second or third if data	7	Amoxicillin and Clarithromycin	*S. pneumoniae* resistance
		Use second line, as resistance to first (but no data on resistance to second)	1	Co-Amoxiclav and Clarithromycin	
		Use second line	10	Co-Amoxiclav and Clarithromycin	
		Use third line (if exists)	24	Levofloxacin	
		Consider alternatives! Resistance to all recommended therapies seen	3		
**Hospital acquired** **pneumonia**	0.74 (0.16)	Use first line	2	Co-Amoxiclav	*S. aureus* and *P. aeruginosa* resistance
		Consider alternatives! Resistance to all recommended therapies seen	43		
**Cellulitis / skin** **abscess**	0.55 (0.20)	Use first line	30	Flucloxacillin	*S. aureus* resistance
		Use first line, but no data to inform – consider second or third if data	6	Flucloxacillin	
		Use second line	2	Clindamycin	
**Bacterial** **meningitis**	0.51 (0.32)	Use first line, but no data to inform – consider second or third if data	10	Penicillin and Gentamicin	*
		Use second line, as resistance to first (but no data on resistance to second)	4	Ceftriaxone and Gentamicin	
**Septic arthritis**	0.57 (0.09)	Use first line but no data to inform – consider second or third if data	15	Oxacillin	*

S.D., standard deviation. *There were little data to establish key drivers.

**Figure 5.**
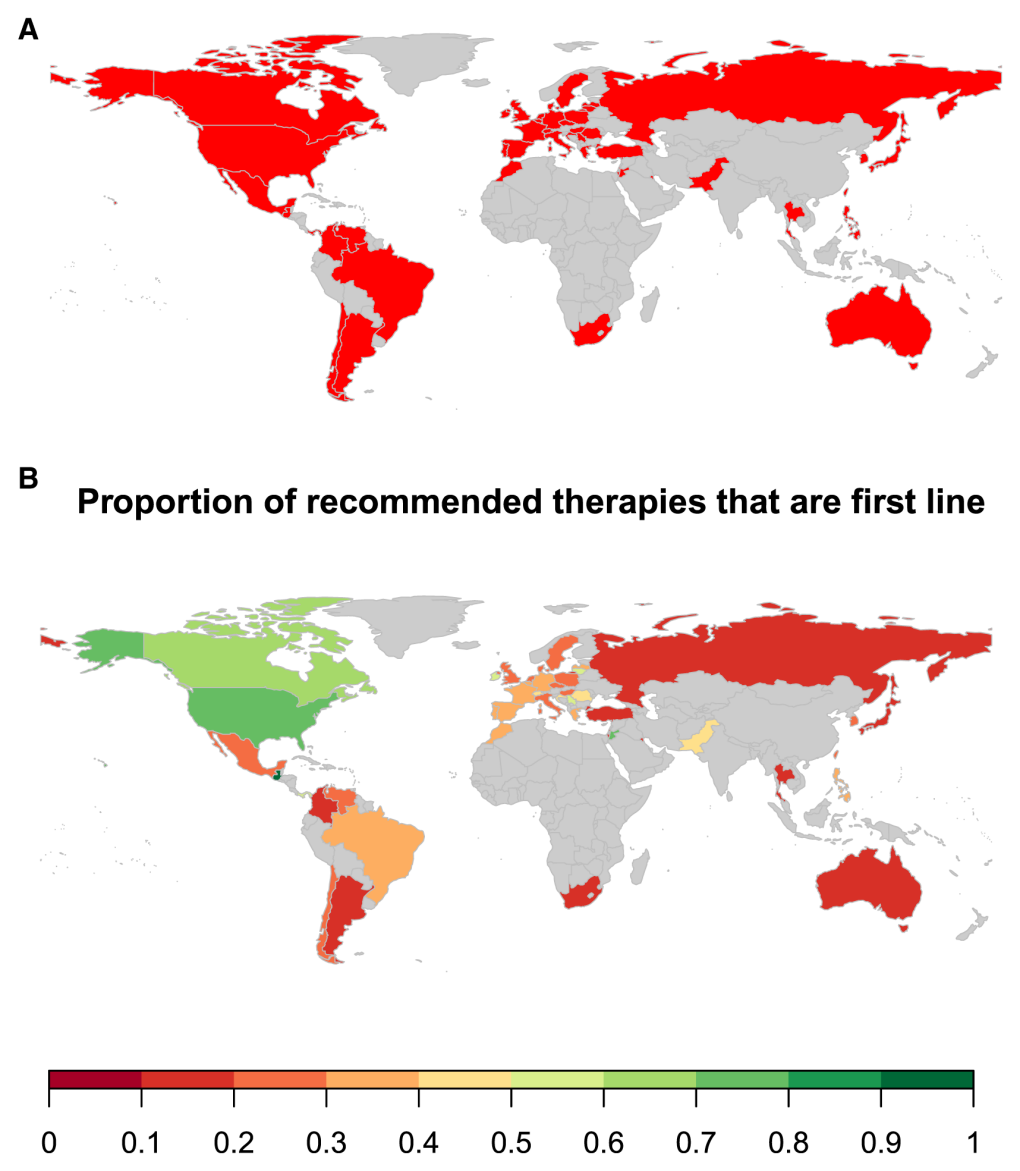
Map of ATLAS data. ( **A**) Countries in red are represented in the subset of the ATLAS dataset used in our analysis. ( **B**) Map of first line therapy proportion. Colours represent the proportion of syndromes for that country that we recommended to use first line therapy. Those in grey had no data for all syndromes.

Applying these criteria resulted in a subset of 435,557 (69%) isolates from the ATLAS dataset that could be used to inform empiric guidelines. When grouped by country, species, syndrome and antibiotic, this resulted in 16,596 data points we could use in the AR.IA App. These data points represent resistance levels to individual antibiotics in our empiric guidelines in species isolated from a syndrome source in a single country. This subset of ATLAS isolates came from 46 countries only ( Figure 5A), out of an original 73, limiting the number of countries we could generate recommendations for.

***(g) Resulting recommendations on the appropriateness of empiric antibiotic therapies***. Recommendations existed for at least one of our nine syndromes for all 46 countries in the merged dataset. On average, each syndrome had recommendations for 32 countries ranging from 14 countries for bacterial meningitis to 44 countries for bloodstream infection ( Table 3). Most of these countries were in Europe, the Americas and Asia, and only two in Africa (South Africa and Morocco) ( Figure 5A). This reflects the underlying availability of isolates in the ATLAS dataset.

First-line antibiotics were recommended if the composite resistance index for the infection syndrome was lower than the default resistance cut-off (15%). This cut-off was chosen because this represented a resistance level recently used for a local change in empirical antibiotic use for treatment of suspected severe infection. It is expected that users might want to use different thresholds for different syndromes, taking into account the trade-off between syndrome severity and other factors including cost and future promotion of antibiotic resistance. If susceptibility data to first line antibiotics was not available, we recommended first line therapy but clarified this as "Use first line, but no data to inform - consider second or third if data".

Most syndromes had a mean causative pathogen availability, across all countries and antibiotics, of above 50% ( Table 3), except for complicated urinary tract infection (UTI) (2%) and upper respiratory tract infection (32.5%)

Empiric therapy recommendations by syndrome derived from the ATLAS dataset are given in Table 3, which lists the recommendations for syndromes with a mean causative pathogen availability of at least 50%. However, the recommendations for bacterial meningitis and septic arthritis rely on our assumption to treat missing susceptibility information as indicating full susceptibility, as indicated by the “no data” mention. Therefore, we consider here that we cannot give robust recommendations for these two syndromes. 

We found that 42/44 countries had resistance to all recommended therapies for the treatment of bloodstream infection. This was driven by high levels of amoxicillin (first-line therapy) resistance in *Staphylococcus aureus* (assumed to cause 25% of bloodstream infection cases). In total, 48% of *S. aureus* bloodstream infection isolates in the ATLAS database were resistant to oxacillin and hence phenotypically MRSA, which are also resistant to cefuroxime (the 2 ^nd^ line agent). The high levels of resistance to first-line antibiotics seen in hospital-acquired pneumonia were driven by high proportions of beta-lactam resistance (cefuroxime in this therapy) in *S. aureus* and *P. aeruginosa* contributing 35% and 28% to this syndrome, respectively. A similar pattern was seen in community-acquired pneumonia, in this case driven by *Streptococcus pneumoniae* resistance to amoxicillin. For cellulitis/skin abscess the opposite was true, with the majority (>75%) of countries being recommended to use the first line antibiotic (flucloxacillin).

*E. coli* causes around 70% of complicated UTI but this species was not commonly tested for trimethoprim susceptibility in the ATLAS dataset, which resulted in a mean causative pathogen availability of only 2% across all countries. Low causative pathogen availability for complicated UTI and respiratory tract infection meant that recommendations could not be strongly supported for these syndromes.

In summary, we could make recommendations for a limited number of infection syndromes using the ATLAS dataset: only for bloodstream infection, pneumonia and cellulitis/skin abscess. Our algorithm frequently recommended use of last-resort therapies due to high levels of resistance in *S. aureus* and *Pseudomonas aeruginosa.* This contrasts with the lower antibiotic resistance rates derived from ResistanceMap, ECDC and GLASS datasets, which suggests that there may be systematic differences in the way these datasets were generated.

Most of our final recommendations included therapies that were in the WHO Essential Medicines List and none were on the AWaRE reserve list. This means that theoretically most of the recommended antibiotics should be available on the market, even in resource constrained settings.

### The AR.IA App

The AR.IA App (available here: https://gwenknight.shinyapps.io/empiric_prescribing/) ^18^ presents the underlying data and recommendations described above. User instructions on how to use the App are presented in the section *AR.IA App Documentation* in *Extended Data* ^38^. The AR.IA App allows the user to choose and combine multiple AMR surveillance datasets when calculating the syndrome-level composite resistance index. It allows the user to change multiple parameters, including syndrome type, resistance cut-off and aetiology. Recommending a change in antibiotic prescribing is a binary decision based on the resistance cut-off, users can change this cut-off to explore changes is antibiotic recommendations when the composite resistance level is close to the resistance cut-off. It can produce visual aids like the maps showed above (see Figure 4B, showing a global map of the proportion of syndromes for each available country for which we still recommended to use first-line therapy).

## Discussion

We aimed to determine whether open-access AMR surveillance datasets, such as the newly available ATLAS dataset, could be used to inform on the appropriateness of empiric antibiotic therapies to treat common infection syndromes. We integrated data on which antibiotics are commonly prescribed as empiric therapy, the bacterial aetiology of each syndrome and the antibiotic susceptibilities of syndrome-contributing bacteria to produce a syndrome-level composite resistance index. We presented our results on an interactive web app, the AR.IA App ^18^, to allow users to explore the impact of resistance proportions on prescribing decisions. Our code is available in an open-access format and broken down into discrete sections that can be re-used and modified by any user. To our knowledge, this is the first time that antibiotic resistance estimates have been compared between multiple global AMR surveillance datasets and linked to the MSH International prices dataset to present a coalition of resistance, proxy cost and proxy access indicators.

Despite the variety of antibiotics tested, clinical sources, bacterial species and countries represented in the ATLAS dataset, we often found there were not enough isolates—from syndrome-causing bacteria, syndrome-relevant sources and tested for the antibiotic of interest—to calculate composite resistance indices for most syndromes. As a result, we could only derive country-level recommendations for relatively few infections (bloodstream infection, pneumonia and cellulitis/skin abscess). We also noted what appeared to be an over-representation of antibiotic-resistant isolates in the ATLAS dataset, as compared to the ECDC and ResistanceMap datasets. While other surveillance datasets typically only include data on the first isolate per patient, mostly from blood and cerebrospinal fluid, the sampling methods for the ATLAS dataset are unclear to us. Our results suggest there may have been a sampling bias in the ATLAS dataset to test for non-susceptible isolates or particular types of infections with higher proportions of resistance. We are therefore more likely to observe resistance to first-line therapies in the ATLAS dataset, which leads to more frequent recommendation of last-line therapies. This is likely to be a common issue in AMR data generated from convenience sampling of clinical databases in settings with limited access to microbiology services. The ideal situation would be universal or representative sampling from all patients with suspected infection. Finally, the relatively low agreement values between ATLAS and the other AMR surveillance highlight the need for critical appraisal before using ATLAS to inform empiric prescribing in its current form. Nevertheless, to the best of our knowledge, the ATLAS dataset will remain freely accessible, and will be updated every 6 to 8 months, which could improve its usefulness to inform empiric prescribing in the future. An added improvement to the dataset would be the conversion of MIC values to sensitive/resistant classifications (e.g. using EUCAST guidelines) – currently there are measurements without classifications. We did not perform this decision making here, in part due to conflicting thresholds in different guidelines and due to the fact that many of the antibiotics with missing classifications are not used for empirical guidelines (e.g. colistin), but this would increase the data available for future analyses.

Our analysis has several limitations. We only included a limited set of infection syndromes and hence used only part of all available ATLAS entries. Future work should include other syndromes, such as purulent urethritis (typically caused by *Neisseria gonorrhoeae*), and sub-classify broad syndromes into narrower types of infections. The aim of this work is to inform prescribing after a decision, based on clinical examination, has been made as to the site and type of infection (i.e. the syndrome). Apart from bloodstream infections, which do often co-exist with other syndromes, our assumption that syndromes were independent is likely to hold but in practice few of the infection syndromes have entirely reliable identification. This simplification of syndromes is a limitation of our work but also of empiric prescribing in general.

Syndrome aetiology was informed only by basic literature reviews and will need to be supported by in-depth systematic reviews and account for regional, seasonal and host population differences. At this stage, we allow AR.IA App users to change the aetiological distributions. The choice of antibiotics used as empiric therapies could also be inputted by the user. The level of recommendations (i.e. country-level) was dictated by the type of sampling available from global AMR surveillance datasets. Increased granularity will be needed to tailor antibiotic prescribing guidelines to local settings (e.g. hospital-level). Alternatively, resistance data could be pooled across multiple neighbouring countries with sparse availability of resistance information.

An important assumption we make is that bacteria with missing antibiotic susceptibility information are considered to be fully susceptible to that antibiotic. If we were to reverse this assumption, and consider that bacteria with missing information are fully resistant, this would push our recommendations towards second or third line therapy, or even to the conclusion that none of the therapies would be effective. As we provide the value of our resistance index for all levels of therapy in the AR.IA App regardless of our final recommendation, users can themselves choose to interpret missing values (indicated by “NA”) as being a synonym of resistance rather than susceptibility, and escalate therapy to the next line.

AMR surveillance datasets do not report the susceptibilities of antibiotics that bacteria are commonly sensitive or intrinsically resistant to. For example, *Pseudomonas aeruginosa* is intrinsically resistant to many beta-lactam antibiotics and thus it is never tested against these agents. AMR surveillance datasets will need to systematically incorporate these rules. Datasets other than ATLAS reported susceptibility to antibiotic classes (e.g. cephalosporins), instead of that to individual drugs (e.g. ceftriaxone), which was not helpful for empiric therapy design as resistance is not always common to all antibiotics belonging to the same class. Relatively low isolate numbers for some countries restricted the potential usefulness of the datasets, and this factor also forced us to rely on potentially outdated numbers from previous years (up to 2015 for ResistanceMap). Additionally, the data presented in these surveillance datasets focus on hospital-associated infections, which limits their relevance for community-associated infections. Given these limitations, these AMR surveillance datasets may not be the most reliable source of AMR proportions to base empirical treatment on. Identifying such an “optimum arrangement for recording and reporting of AMR data” is indeed one of the objectives of the UK Five-Year Antimicrobial Resistance Strategy ^40^. This includes using point prevalence surveys as the gold standard (as opposed to convenience sampling of isolates from clinical specimens that may be biased towards resistant strains) to estimate the total burden of AMR, determine the aetiology of common infection syndromes and ultimately inform empiric antibiotic guidelines.

This work focuses on improving guidelines for empiric treatment of infection, but there are limitations to the usefulness of this approach ^41^. Firstly, individual patients identified by laboratory investigation to have antibiotic-susceptible infections can still be effectively treated with agents that show extensive resistance proportions at the population level. Secondly, empiric guidelines typically favour the use of broad-spectrum antibiotics to achieve the highest chance of treatment success, but this may not always be in the individual or population’s best overall interest in terms of minimizing side-effects (such as *Clostridium difficile* infection) or conserving effectiveness of treatments. Thirdly, some drugs with low-levels of resistance but multiple other sub-optimal properties (such as vancomycin) may be recommended in later lines of empiric therapy at high levels of resistance. And fourthly, the assumption is made that infection syndromes are caused entirely by bacterial pathogens requiring antibiotic treatment, when in reality many infections are caused by viruses ^42^ and would recover without needing antibiotics. Whilst future advances on rapid and cheap point-of-care diagnostics for AMR bacteria might remove the need for empiric therapy, these will continue to widely be used in many settings, especially in low-income countries.

Future iterations of this app should include user defined therapies as well as the option to upload local resistance data. We also had a binary resistance cut-off. This means that, for example, if the cut-off is set to 15% (and resistance to first line antibiotics is 16%), even if resistance to second-line antibiotics is 14% they will still be recommended. For now, users who suspect this may be an issue can alter this cut-off and see its effects on the recommendations (for example switch to 14% manually and see if there is a change in recommendation). Future iterations should include a range around the cut-off which would produce suitable warnings. 

## Conclusion

We have shown how independent sources of data can be combined with AMR surveillance information, such as the ATLAS dataset, to add clinical and policy-making value. Our results suggest that whilst the creation of a composite resistance index is technically feasible, the data needed to make robust prescribing recommendations for most infectious syndromes is currently lacking. In line with a move towards more evidence-based antibiotic prescribing, we believe this approach could be used to monitor the effectiveness of antibiotic empiric therapies, the cornerstone of current antibiotic prescribing practices. Such an approach can be applied to more robust data as these become available.

## Data availability

### Underlying data

Table 4 contains the underlying data used in this study; Table 5 contains these data compiled for use in the AR.IA App.

**Table 4.** Source of the underlying data used in this study.

Title	Use	Reference
ATLAS	Source of laboratory data	https://atlas-surveillance.com/#/login and https://amr.theodi.org/programmes/atlas
ECDC Surveillance Atlas	Used in comparison of datasets	Dataset provided by ECDC based on data provided by WHO and Ministries of Health from the affected countries: https://ecdc.europa.eu/en/antimicrobial-resistance/surveillance-and- disease-data/data-ecdc
ResistanceMap	Used in comparison of datasets	The Center for Disease Dynamics Economics and Policy. ResistanceMap: Antibiotic Resistance. 2018. https://resistancemap.cddep.org/AntibioticResistance.php. Date accessed: February 13, 2019.
GLASS	Used in comparison of datasets	Dataset provided by the World Health Organization. https://www.who.int/glass/en/
MSH International Products Price Guide	Price of drug and placement on WHO Essential Medicines list	Management Sciences for Health. The International Medical Products Price Guide (2015 Edition) (2016).
World Bank CPI	Inflation of drug costs	The World Bank. Inflation, consumer prices (annual %). (2019) https://data.worldbank.org/ indicator/FP.CPI.TOTL.ZG [Accessed 20/02/2019]
AWaRe	Placement on AWaRe list	Sharland, M., Pulcini, C., Harbarth, S., Zeng, M., Gandra, S., Mathur, S. and Magrini, N. Classifying antibiotics in the WHO Essential Medicines List for optimal use—be AWaRe. The Lancet Infectious Diseases, 18(1), pp.18–20. (2018). http://apps.who.int/medicinedocs/ documents/s23413en/s23413en.pdf
NICE guidelines	Empiric antibiotic therapies	The National Institute for Health and Care Excellence (NICE) and Public Health England (PHE). Summary of antimicrobial prescribing guidance - managing common infections. 1–23 (2019): https://www.nice.org.uk/Media/Default/About/what-we-do/NICE-guidance/

**Table 5.** Data from Table 4 compiled for use in the final running of the AR.IA App.

Title	DOI	Use	Reference
AR.IA App all datasets	https://doi.org/10.6084/m9.figshare.9821996	Data from the ATLAS dataset, the ECDC Surveillance Atlas, ResistanceMap, and GLASS aggregated by species and drugs of interest in our analysis	43
AR.IA App drug breakdown	https://doi.org/10.6084/m9.figshare.9822077	Baseline empiric therapy recommendations	44
AR.IA App drug breakdown (groups)	https://doi.org/10.6084/m9.figshare.9822104	Baseline empiric therapy recommendations with drug groupings instead of individual drugs	45
AR.IA App species breakdown	https://doi.org/10.6084/m9.figshare.9822179	Baseline contributing bacteria distributions	46
AR.IA App all species	https://doi.org/10.6084/m9.figshare.9822146	List of species to include	47
AR.IA App economic data	https://doi.org/10.6084/m9.figshare.9822122	Cost, WHO EML and AWaRe	48

### Extended data

Figshare: AR.IA paper Extended Data - Further Methods. https://doi.org/10.6084/m9.figshare.9852029.v2 ^34^.

This project contains further information on the manipulation of the ATLAS dataset, the comparison of the ATLAS dataset with ECDC Surveillance Atlas and ResistanceMap, the creation of our drug-resistance index, and details on other drug information datasets used in our analysis.

Figshare: AR.IA paper Extended Data - Further Results. https://doi.org/10.6084/m9.figshare.9852041.v2 ^39^.

This project contains further information on the comparison of the ATLAS dataset with ECDC Surveillance Atlas and ResistanceMap, the antibiotics used in our analysis, the summary of our review for the aetiology of the infection syndromes, the process to combine the antimicrobial resistance datasets, and to combine the drug information datasets. Also contained are Supplementary Figures 1–3, and Supplementary Tables 1–6.

Figshare: AR.IA paper Extended Data - AR.IA App documentation. https://doi.org/10.6084/m9.figshare.9852056.v2 ^38^.

This project contains instructions regarding the usage of the App developed as part of this project. Also contained is Supplementary Figure 4.

Extended data are available under the terms of the Creative Commons Attribution 4.0 International license (CC-BY 4.0).

## Software availability

The AR.IA App is available at: https://gwenknight.shinyapps.io/empiric_prescribing/.

Source code available from: https://github.com/gwenknight/empiricprescribing.

Archived source code at time of publication: https://doi.org/10.5281/zenodo.3418998 ^18^.

Licence: GNU General Public License v3.0.
