# Supplementary material for: Feasibility of informing syndrome-level empiric antibiotic recommendations using publicly available antibiotic resistance datasets
Source: Wellcome Open Res. 2020 Jun 24;4:140. Originally published 2019 Sep 24. [Version 2] doi: 10.12688/wellcomeopenres.15477.2 (PMC7327722; doi:10.12688/wellcomeopenres.15477.2)
Supplement: Peer review history

# Peer review: Reviewer response for version 2

**DOI:** 10.21956/wellcomeopenres.17655.r39220

**Published:** 2020-06-30

**Authors:** Liam Shaw

**Affiliations:** 1 Nuffield Department of Clinical Medicine, University of Oxford, Oxford, UK

Thanks to the authors for their revisions and responses. I'm happy to approve the manuscript in its revised form.

Is the work clearly and accurately presented and does it cite the current literature?

Partly

If applicable, is the statistical analysis and its interpretation appropriate?

Yes

Are all the source data underlying the results available to ensure full reproducibility?

Yes

Is the study design appropriate and is the work technically sound?

Yes

Are the conclusions drawn adequately supported by the results?

Yes

Are sufficient details of methods and analysis provided to allow replication by others?

Yes

Reviewer Expertise:

My main research area is bacterial genetics, including antimicrobial resistance. I also have direct experience of analysing the ATLAS dataset used in this work.

I confirm that I have read this submission and believe that I have an appropriate level of expertise to confirm that it is of an acceptable scientific standard.

# Peer review: Reviewer response for version 2

**DOI:** 10.21956/wellcomeopenres.17655.r39218

**Published:** 2020-06-30

**Authors:** Marlieke de Kraker

**Affiliations:** 1 Infection Control Program, Faculty of Medicine, World Health Organization Collaborating Centre on Patient Safety (Infection Control and Improving Practices), University of Geneva Hospitals, Geneva, Switzerland

I thank the authors for the responses, and I approve the current version of the manuscript.

Is the work clearly and accurately presented and does it cite the current literature?

Partly

If applicable, is the statistical analysis and its interpretation appropriate?

Partly

Are all the source data underlying the results available to ensure full reproducibility?

Yes

Is the study design appropriate and is the work technically sound?

Yes

Are the conclusions drawn adequately supported by the results?

Partly

Are sufficient details of methods and analysis provided to allow replication by others?

Yes

Reviewer Expertise:

I am an epidemiologist working in the field of antimicrobial resistance, and I have ample experience with AMR surveillance data.

I confirm that I have read this submission and believe that I have an appropriate level of expertise to confirm that it is of an acceptable scientific standard.

# Peer review: Reviewer response for version 1

**DOI:** 10.21956/wellcomeopenres.16928.r36746

**Published:** 2020-02-25

**Authors:** Ben S. Cooper

**Affiliations:** 1 Nuffield Department of Medicine, University of Oxford, Oxford, UK

This feasibility study represents a valuable addition to the surprisingly small literature on how to make empiric antibiotic recommendations. It is clearly written and well-organised, and the framework presented describes a sensible approach to using existing data-sets. Limitations of the approach, most of which are related to current limitations in publicly available data-sets, are clearly acknowledged. Suggestions for improvements below are mostly relatively minor, though points 5 and 6 below seem important to address.

1. Page 4, paragraph 2. “Concordance” would be a better word than “accuracy here” as there is no gold standard surveillance system. “Accuracy” is also used elsewhere in the manuscript.
2. Figure 1. Can the caption explain what the direction of the arrows indicates?
3. Page 5 “…which is set to 15% by default”. What is the reason for choosing 15%? Perhaps this point could be considered in the discussion. Surely it would make sense to choose different cut-offs for different syndromes as the trade-off between impact on patient outcomes and future selection for resistance will be different. 
4. Page 5 “missing isolates”. I don’t think I understand why the term “missing isolates” is used. Were the isolates missing or were the records just incomplete?
5. Figure 3: I don’t completely understand the logic of this. If there are no data on the first line therapy wouldn’t it make sense to make no recommendation but to collect more data, rather than immediately going on and recommend the second line therapy.
6. Table 3: Some of the recommendations here seem incompatible with Figure 3. e.g the first row for bacteraemia recommends using second line, but says no data on resistance to second line, while in the flow chart (Fig 3) the second line can only be recommended if resistance level is below the cut-off. Similar issues for septic arthritis. It would also be useful to have some indication of the strength of the evidence for the recommendation in the table. 
7. Page 9, paragraph 2. Should make it clear that the cut-off of 15% can be changed in the app.

Is the work clearly and accurately presented and does it cite the current literature?

Yes

If applicable, is the statistical analysis and its interpretation appropriate?

Yes

Are all the source data underlying the results available to ensure full reproducibility?

Yes

Is the study design appropriate and is the work technically sound?

Yes

Are the conclusions drawn adequately supported by the results?

Yes

Are sufficient details of methods and analysis provided to allow replication by others?

Yes

Reviewer Expertise:

Communicable disease epidemiology & antimicrobial resistance.

I confirm that I have read this submission and believe that I have an appropriate level of expertise to confirm that it is of an acceptable scientific standard, however I have significant reservations, as outlined above.

# Response

**Published:** 2020-06-19

**Authors:** Quentin Leclerc

**This feasibility study represents a valuable addition to the surprisingly small literature on how to make empiric antibiotic recommendations. It is clearly written and well-organised, and the framework presented describes a sensible approach to using existing data-sets. Limitations of the approach, most of which are related to current limitations in publicly available data-sets, are clearly acknowledged. Suggestions for improvements below are mostly relatively minor, though points 5 and 6 below seem important to address.**

 Thank you for your comments - we are glad you found it a valuable addition. We have left your comments in bold and italics below, while our answers are written in plain text.

1. **Page 4, paragraph 2. “Concordance” would be a better word than “accuracy here” as there is no gold standard surveillance system. “Accuracy” is also used elsewhere in the manuscript.**

Thank you for this suggestion. In light of other reviewer comments, we have decided to replace “accuracy” by “agreement” throughout the manuscript. We hope that this is a satisfactory change.

1. **Figure 1. Can the caption explain what the direction of the arrows indicates?**

We have now clarified in the caption that the arrows linking the boxes indicate the process of integrating the different open-access datasets together to generate our index to inform empiric therapy. In addition, following suggestions from other reviewers, we have modified the figure to include two types of arrows to improve readability.

1. **Page 5 “…which is set to 15% by default”. What is the reason for choosing 15%? Perhaps this point could be considered in the discussion. Surely it would make sense to choose different cut-offs for different syndromes as the trade-off between impact on patient outcomes and future selection for resistance will be different.  **

Such a cut-off is always going to be a somewhat arbitrary choice – we used 15% as this value had recently been used in the clinical practice for a similar question for one of us. In other situations, we have found that other cut-off values have been used – for example 25% for treatment of pneumonia in one recommendation from USA (see reference in text). We agree that different cut-offs would probably selected in different circumstances by different users for different syndromes – hence the value of having a slider for this parameter in the app. However, in the main text, for simplicity we chose to use just one value (15%).

1. **Page 5 “missing isolates”. I don’t think I understand why the term “missing isolates” is used. Were the isolates missing or were the records just incomplete?**

Thank you for spotting this; we have corrected this phrase to indicate that the isolates were indeed not missing, but had incomplete records (MIC values were missing the corresponding susceptibility labels).

1. **Figure 3: I don’t completely understand the logic of this. If there are no data on the first line therapy wouldn’t it make sense to make no recommendation but to collect more data, rather than immediately going on and recommend the second line therapy.**

Thank you for highlighting this – there was a mistake in this figure in that we do currently recommend first line therapy if there is no data. In the app recommendations, where relevant data is missing, we have:

- "Use second line, as resistance to first (but no data on resistance to second)"
- "Use third line, as resistance to both first and second (but no data on resistance to third)"
- "Use first line, but no data to inform - consider second or third if data"

Where there is resistant to the lower rank therapy, then we recommend use of the next level – “Use second line” in this app is purely because there is resistance to first line. Figure 3 has now been updated to better reflect the logic we use in our recommendations, and highlight that we assume bacteria are susceptible if there are no data to inform on potential resistance.

 The aim of our work was to see what recommendations could be made at the empiric prescribing level by deriving a syndrome-level index. Therefore, we are interested in the immediate decision a prescriber would make solely based on this index. You are correct when saying that ideally, more data would have to be collected before making any decision. However, we assume that we are in a situation where a patient requires immediate antibiotic treatment, so we report what the app recommends given this set of data. In line with comments from another reviewer we have included more discussion to highlight our focus on this secondary part of the empiric prescribing process.

                

1. **Table 3: Some of the recommendations here seem incompatible with Figure 3. e.g the first row for bacteraemia recommends using second line, but says no data on resistance to second line, while in the flow chart (Fig 3) the second line can only be recommended if resistance level is below the cut-off. Similar issues for septic arthritis. It would also be useful to have some indication of the strength of the evidence for the recommendation in the table. **

Thank you for spotting this; as discussed in response to your comment 5), there was an error in Figure 3, which has now been corrected. As for an indication of the strength of the evidence, we have not mentioned this in Table 3 as it would vary between countries, but in the AR.IA App we now indicate the number of isolates used to generate the recommendation as a measure of strength of evidence. 

1. **Page 9, paragraph 2. Should make it clear that the cut-off of 15% can be changed in the app.**

Thank you for highlighting this. We did already mention that the cut-off could be changed in the app in the “Methods, The AR:IA App creation” section, however we have added another phrase in the paragraph you mentioned to hopefully make it clearer that we were referring to the 15% cut-off.

# Peer review: Reviewer response for version 1

**DOI:** 10.21956/wellcomeopenres.16928.r37548

**Published:** 2020-02-05

**Authors:** Marlieke de Kraker

**Affiliations:** 1 Infection Control Program, Faculty of Medicine, World Health Organization Collaborating Centre on Patient Safety (Infection Control and Improving Practices), University of Geneva Hospitals, Geneva, Switzerland

**General comments**

 I would like to congratulate the authors with this original, comprehensive article. It describes the development of a freely available app to provide an evidence base for syndrome- and country-specifc empirical prescribing. The resistance data is derived from four different datasets, including one commercial dataset, ATLAS, and three public datasets, EARS-Net, ResistanceMap and GLASS. This research was carried out as part of the Wellcome Data Re-use Prize, promoting the use of pharmaceutical AMR data.

 While it is an interesting tool, part of the objective of this study was to look at the applicability of ATLAS data. The authors mention that the resistance proportions reported by ATLAS are higher than those reported by the other datasets. I would have liked to know more about the ATLAS database in order to understand these differences and avoid misuse in the future. For example, the type of samples collected (colonization, infection, community, hospital), the sampling strategy (first isolate per species, per patient, all isolates or only those with susceptibility results for last-resort antibiotics, i.e. with confirmed resistance to first-line antibiotics), method of antimicrobial susceptibility testing (central, local, automated, applied breakpoints etc.) etc.

 Based on the discrepancies they found between ATLAS and the public datasets, I think the app should have a warning indicating that the reported data are not necessarily representative, and mainly based on samples from the hospital setting. This should also be emphasized in the conclusion of the abstract and article.

 The app could also have been further improved by indicating the number of isolates that the advice was based on per country so users can appreciate the validity of the data for their setting.

**Detailed comments**

**Abstract**

 The introduction lacks a clear objective, which seems to be included in the Methods section “ to derive .. syndromes” . I suggest to move this to the introduction and make the objective PICO.

 The Methods section could explicitly mention the other datasets, and provide a bit more detail of what was done.

 In the Results section, I would like to see some explicit results, like the number of isolates and countries that were available to inform the system. The specific syndromes that could be selected, etc.

 In the conclusions, “rates of AMR” is used I think “AMR proportions” is more correct.

**Introduction**

 In paragraph 4, it is described that “Providing .. guidelines based on open acces international AMR surveillance data may help practitioners”. I think what is meant is providing a tool, not guidelines to help inform empirical prescribing.

 In the last paragraph it is mentioned that “We propose a more clinically-oriented presentation of resistance rates” and “we designed a syndrome-level composite resistance index”. This suggests that this is a novel idea of the authors, but as described in the discussion such a kind of index has already been proposed by multiple others. If I understood correctly, the Weighted Incidence Syndromic Combination is exactly the same. ^1^ I suggest to move the paragraph about these indices from the discussion to the introduction, and make it clear that this is not a novel concept, but that this concept is now taken further by developing an easy-to-use tool informed by publicly available resistance data.

**Methods**

**Surveillance data comparison**

 It is stated that “The ATLAS dataset .. contains high-quality AST data”. However, it is not explained why it is considered “high-quality”. Please elaborate.

 Could you add an address where the ATLAS data can be found for interested researchers?

 Please report about the sample selection procedure for all datasets, as this heavily influences resistance proportions. For the surveillance systems it is commonly the first isolate per sample type per patient per year, and as they focus on blood samples all are clinical samples, mainly from the hospital setting. It would be important to have the same information for the ATLAS dataset.

 The type of variables reported to ATLAS would be informative as well. In the app you can select age groups, for example, but availability of this data has never been discussed in the paper.

 Please add the type and year of publication of the breakpoints that were applied to MIC data with missing S/I/R interpretation.

 In the fifth paragraph, “resistance rates” are often referred to, this should be “resistance proportions”.

 Here it is also stated that “ ECDC and ResistanceMap .. derive from the same source data”. I think it is more correct to state that ResistanceMap includes all ECDC data, and uses other sources for AMR data from other continents. Moreover, the same can be explained for GLASS, all European sepsis data is derived from ECDC data.

 “Accuracy” might not be the best term, perhaps “agreement” is more clear.

 Step c) Why was additional clinical consultation needed, and how were the NICE guidelines and the clinical consultation integrated?

 Step g) I think “syndrome coverage” can be explained a bit more clearly. Perhaps another term should be used, like “causative pathogen availability”.

 Step g) If data is missing full susceptibility is assumed. This is a strong assumption, which would have warranted a sensitivity analysis for a best-case worst-case scenario, which could even have been incorporated in the app.

**Results**

 In table 1, and supplementary figures 2 and 3, please report the number of countries included in each dataset.

 In table 1, I would also like to see the total number of isolates per dataset, and the overlap in countries between datasets clearly displayed.

 Figure 1. I think the file names can be removed. “Other datasets” could be replaced by the real names, including ATLAS.

 Figure 3. The arrow from “Are there data on first-line therapy - No”, results in second line therapy being suggested (if data is available). This does not seem to be in line with what is discussed in step g) where missing data is assumed “fully susceptible” please align.

 Figure 4. I would not give the same (grey) colour to countries that had no data, or had no syndrome for which first-line therapy was recommended. These are two, very distinctive categories.

**Surveillance data comparison**

 The “accuracy” here is shockingly low, it would have been interesting to see whether certain specific bug-drug combinations were responsible for the difference in AMR proportions, and how large the differences in AMR proportions were. The proportion of overlap by itself is not very informative.

**Step e)**

 How does it work if the datasets are combined? Are all isolates just aggregated without any weighting? And if you switch between only showing ATLAS data, or showing data from 2 or more datasets including ATLAS are ATLAS data then transformed to class resistance versus single antibiotic resistance when only ATLAS is selected? Please clarify.

 How much overlap was there between the datasets in country coverage? It would have been nice to have maps indicating country participation for the different datasets.

 While you mention that you could only provide recommendations for bacteremia, pneumonia and cellulitis, in the app and table 3, meningitis and arthritis are still included?

**App**

 I think a line should be included explaining that the included AMR proportions are from hospital-associated infections. And a warning should be included that the data may not be representative of the national prevalence of AMR due to selective sampling (mainly tertiary care hospital data, selection of isolates with full AST data, i.e. the more resistant ones, etc.).

 The tool could have been even more useful if uploading of local resistance data was made easy. The possibility to export country-specific data could be helpful too. If this is easy to add, I would like to ask the authors to add this functionality, if it is a lot of work this would not be required.

 The cut-off for selecting first or second line antibiotics is binary which means that second line therapeutics could be advised even if resistance proportions only differ 1% point. Could it somehow be incorporated that only above a clinically relevant difference in resistance the advice would switch from first to second line antibiotics? As above, if this is easy to add, I would like to ask the authors to add this functionality, if it is a lot of work this would not be required. Then, I would like to see this remark added to the paper.

**Discussion**

 In the limitations section, it should be discussed that these data may not be the most reliable source of AMR proportions to base empirical treatment on, especially since for certain countries the number of isolates included are very low and not very up-to-date (2017).

 It should also be mentioned that the provided data focus on hospital-associated infections (and tertiary care centres) and do not apply to community-associated infections.

 Finally, there should be a warning against the future use of ATLAS data without critical appraisal; there is very low “accuracy”, and too little information about the sampling scheme and its representativeness. On certain websites ( https://www.amrindustryalliance.org/case-study/antimicrobial-testing-leadership-and-surveillance-atlas/) they claim that ”ATLAS is the only database of its kind capable of providing such a broad scope of reliable, readily available information in an easy to use platform” which is “crucial for clinicians and public health officials”, which could have important implications.

 Please also report about the future prospects of ATLAS data, and whether this database will remain available and/or will be updated with new data?

Is the work clearly and accurately presented and does it cite the current literature?

Partly

If applicable, is the statistical analysis and its interpretation appropriate?

Partly

Are all the source data underlying the results available to ensure full reproducibility?

Yes

Is the study design appropriate and is the work technically sound?

Yes

Are the conclusions drawn adequately supported by the results?

Partly

Are sufficient details of methods and analysis provided to allow replication by others?

Yes

Reviewer Expertise:

I am an epidemiologist working in the field of antimicrobial resistance, and I have ample experience with AMR surveillance data.

I confirm that I have read this submission and believe that I have an appropriate level of expertise to confirm that it is of an acceptable scientific standard, however I have significant reservations, as outlined above.

## References

[1] : Weighted-incidence syndromic combination antibiograms to guide empiric treatment of critical care infections: a retrospective cohort study. *Crit Care*.2014;18(3) : 10.1186/cc13901 R112 10.1186/cc13901 24887215PMC4075242

# Response

**Published:** 2020-06-19

**Authors:** Quentin Leclerc

***I would like to congratulate the authors with this original, comprehensive article. It describes the development of a freely available app to provide an evidence base for syndrome- and country-specifc empirical prescribing. The resistance data is derived from four different datasets, including one commercial dataset, ATLAS, and three public datasets, EARS-Net, ResistanceMap and GLASS. This research was carried out as part of the Wellcome Data Re-use Prize, promoting the use of pharmaceutical AMR data.***

 Many thanks for your comments. We have left your comments in bold and italics below, while our answers are written in plain text.

***While it is an interesting tool, part of the objective of this study was to look at the applicability of ATLAS data. The authors mention that the resistance proportions reported by ATLAS are higher than those reported by the other datasets. I would have liked to know more about the ATLAS database in order to understand these differences and avoid misuse in the future. For example, the type of samples collected (colonization, infection, community, hospital), the sampling strategy (first isolate per species, per patient, all isolates or only those with susceptibility results for last-resort antibiotics, i.e. with confirmed resistance to first-line antibiotics), method of antimicrobial susceptibility testing (central, local, automated, applied breakpoints etc.) etc.***

***Based on the discrepancies they found between ATLAS and the public datasets, I think the app should have a warning indicating that the reported data are not necessarily representative, and mainly based on samples from the hospital setting. This should also be emphasized in the conclusion of the abstract and article.***

 We agree that we are relying on a database where the collection methods are unclear but believe it to be a convenience sample of isolates taken from hospital surveillance systems with a bias towards sampling from longer and more complex infections, that are proving harder to treat. We believe this to be the case for most AMR databases with isolate samples including the WHO GLASS surveillance. We need better surveillance with pro-active sampling including of those infections that are not complex and do respond to treatment to understand syndrome aetiology and to get a handle on the overall burden of AMR.

 We found it difficult to add more to our abstract and believe that highlighting the use of ATLAS there then allows readers to move to understanding further what ATLAS is within the paper. We have added further discussion in the discussion section to what we believe the samples in ATLAS and the wider AMR datasets represent.

***The app could also have been further improved by indicating the number of isolates that the advice was based on per country so users can appreciate the validity of the data for their setting.***

 We thank the reviewer for this suggestion – we now report the number of isolates in the Therapy Recommendations tab in the app.

**

***Detailed comments***

**

***Abstract***

***The introduction lacks a clear objective, which seems to be included in the Methods section “ to derive .. syndromes” . I suggest to move this to the introduction and make the objective PICO.***

***The Methods section could explicitly mention the other datasets, and provide a bit more detail of what was done.***

***In the Results section, I would like to see some explicit results, like the number of isolates and countries that were available to inform the system. The specific syndromes that could be selected, etc.***

 Thank you for these suggestions to improve the Abstract. We have tried to incorporate as much of the requested information as possible, while respecting the 300 words limit.

***In the conclusions, “rates of AMR” is used I think “AMR proportions” is more correct.***

 We have now made the recommended change from “rates” to “proportions”.

**

***Introduction***

***In paragraph 4, it is described that “Providing .. guidelines based on open access international AMR surveillance data may help practitioners”. I think what is meant is providing a tool, not guidelines to help inform empirical prescribing.***

 Thank you for spotting this mistake, we have now corrected it since we indeed meant “tool” here.

***In the last paragraph it is mentioned that “We propose a more clinically-oriented presentation of resistance rates” and “we designed a syndrome-level composite resistance index”. This suggests that this is a novel idea of the authors, but as described in the discussion such a kind of index has already been proposed by multiple others. If I understood correctly, the Weighted Incidence Syndromic Combination is exactly the same.*** ***^1^*** ***I suggest to move the paragraph about these indices from the discussion to the introduction, and make it clear that this is not a novel concept, but that this concept is now taken further by developing an easy-to-use tool informed by publicly available resistance data.***

 Thank you for this suggestion. We have moved this discussion paragraph to the penultimate paragraph of the introduction. We have clarified in the last paragraph of the introduction that the novelty of our framework comes from using open-access datasets and presenting the results in an online tool.

**

***Methods***

***Surveillance data comparison***

***It is stated that “The ATLAS dataset .. contains high-quality AST data”. However, it is not explained why it is considered “high-quality”. Please elaborate.***

 Many thanks for this comment, we have clarified this by changing the wording of this sentence.

***Could you add an address where the ATLAS data can be found for interested researchers?***

 The link to the official ATLAS website ( https://atlas-surveillance.com) is already provided as a hyperlink when clicking on “ATLAS dataset” at the beginning of the Methods section. However, we have now added a second link ( https://amr.theodi.org/programmes/atlas) to facilitate download of the ATLAS dataset. These links are also present in Table 4 in the “Data availability” section.

***Please report about the sample selection procedure for all datasets, as this heavily influences resistance proportions. For the surveillance systems it is commonly the first isolate per sample type per patient per year, and as they focus on blood samples all are clinical samples, mainly from the hospital setting. It would be important to have the same information for the ATLAS dataset.***

 The types of samples for each dataset are presented in Table 1. We have also added a sentence discussing this difference in sampling between AMR surveillance datasets in the second paragraph of the Discussion section.

***The type of variables reported to ATLAS would be informative as well. In the app you can select age groups, for example, but availability of this data has never been discussed in the paper.***

 Thank you for pointing this out; we have now added two sentences highlighting that the ATLAS dataset also contains information on gender, age and specimen source in the second paragraph of the Methods section.

***Please add the type and year of publication of the breakpoints that were applied to MIC data with missing S/I/R interpretation.***

 We did not apply external data on breakpoints to the MIC data with missing S/I/R interpretation. Rather, we chose to look for other isolates within the ATLAS dataset of the same bacterial species and MIC which had a susceptibility label, and applied the same label to the isolates which only had the raw MIC values (for details, please refer to our Further Methods in Extended Data).

 To make this clearer to the reader, we have modified the last sentence of the 2 ^nd^ paragraph of the Methods – Surveillance data comparison section to:

 “Missing susceptibility labels (i.e. “resistant”, “intermediate” or “susceptible”) in the ATLAS dataset (443,899/633,820) were assigned from available MIC data for other isolates within the ATLAS dataset. We did not use any external data on breakpoints to derive susceptibility labels for MICs which were not labelled elsewhere within the ATLAS dataset (see Further Methods in Extended Data for details).”

 In addition, we have added a sentence after the third paragraph of our Further Methods to elaborate on our decision to not use external breakpoints data.

***In the fifth paragraph, “resistance rates” are often referred to, this should be “resistance proportions”.***

 Thank you for this suggestion, we have now replaced “resistance rates” by “resistance proportions” throughout the manuscript.

***Here it is also stated that “ ECDC and ResistanceMap .. derive from the same source data”. I think it is more correct to state that ResistanceMap includes all ECDC data, and uses other sources for AMR data from other continents. Moreover, the same can be explained for GLASS, all European sepsis data is derived from ECDC data.***

 We have now added a sentence in paragraph 4 to state that ECDC data is included in ResistanceMap and GLASS.

***“Accuracy” might not be the best term, perhaps “agreement” is more clear.***

 Thank you for this suggestion, we have now replaced “accuracy” by “agreement” throughout the manuscript.

***Step c) Why was additional clinical consultation needed, and how were the NICE guidelines and the clinical consultation integrated?***

 In this first iteration of the app. we needed to decide upon a simple set of empiric antibiotic guidelines with one choice for each infection syndrome, ignoring for now the complexities of pregnancy, age and allergies. For this we took the NICE guidelines as a starting point and then used clinical consultation to give the most commonly used antibiotic treatment. For this we relied on our clinical author’s (AA) experience and overview of what happens in several UK Trusts. We have modified the paragraph to reflect this and hope in future iterations to allow for user input to avoid this reliance on one possible therapy.

***Step g) I think “syndrome coverage” can be explained a bit more clearly. Perhaps another term should be used, like “causative pathogen availability”.***

 Thank you for this suggestion, we have replaced “syndrome coverage” by “causative pathogen availability” throughout the manuscript.

***Step g) If data is missing full susceptibility is assumed. This is a strong assumption, which would have warranted a sensitivity analysis for a best-case worst-case scenario, which could even have been incorporated in the app.***

 We agree that this is a strong assumption. The consequence of this reverse assumption would be to push therapies towards second or third line, or even to the recommendation “Consider alternatives! Resistance to all recommended therapies seen”. To better highlight this, and make sure that the reader understands this assumption and the consequence of reversing it, we have added a paragraph on this topic in the section about limitations in the Discussion, and reworked Figure 4 to clearly show this assumption in the decision-making process.

***Results***

***In table 1, and supplementary figures 2 and 3, please report the number of countries included in each dataset.***

 We have now added this information in Table 1. However, we have not added this to Supplementary Figures 2 and 3 as we believe this would make the Figures harder to read (as the number of countries in each dataset each year can change, and we would therefore need to add a number for every year).

***In table 1, I would also like to see the total number of isolates per dataset, and the overlap in countries between datasets clearly displayed.***

 We have now added a footnote in Table 1 to indicate that ResistanceMap and GLASS use ECDC data for their European blood samples, as well as the total sample size per dataset. Please note that this sample size corresponds to the total number of unique bacteria and drug measurements made, rather than number of isolates (i.e. number of lines) in the ATLAS dataset. This is the definition we also use when comparing sample size in our Further Results, to align with the measures provided in other surveillance datasets. We have clarified this in the Surveillance data comparison section in Methods.

***Figure 1. I think the file names can be removed. “Other datasets” could be replaced by the real names, including ATLAS.***

 This has now been adapted in the Figure to match the suggestions given.

***Figure 3. The arrow from “Are there data on first-line therapy - No”, results in second line therapy being suggested (if data is available). This does not seem to be in line with what is discussed in step g) where missing data is assumed “fully susceptible” please align.***

 Thank you for spotting this mistake. Figure 3 has now been updated to better reflect the logic used the app, and the fact that we assume missing data is “fully susceptible”.

***Figure 4. I would not give the same (grey) colour to countries that had no data, or had no syndrome for which first-line therapy was recommended. These are two, very distinctive categories.***

 Apologies, this was an error in the figure and accompanying legend. Grey now only represents countries that had no data. The colour scheme has been updated to better align with other figures, countries with no syndrome for which first-line therapy was recommended would now be in dark red.

***Surveillance data comparison***

***The “accuracy” here is shockingly low, it would have been interesting to see whether certain specific bug-drug combinations were responsible for the difference in AMR proportions, and how large the differences in AMR proportions were. The proportion of overlap by itself is not very informative.***

 We have now also changed the phrasing of accuracy to agreement in line with previous reviewer suggestions. We agree that such a question would be worth investigating, however we feel that such an in-depth comparison would stand as an entirely different piece of work from our own analysis here, beyond the scope of this paper.

***Step e)***

***How does it work if the datasets are combined? Are all isolates just aggregated without any weighting? And if you switch between only showing ATLAS data, or showing data from 2 or more datasets including ATLAS are ATLAS data then transformed to class resistance versus single antibiotic resistance when only ATLAS is selected? Please clarify.***

 We have indicated in step e) that we aggregated data across multiple datasets “by mapping antibiotics in the ATLAS dataset to their corresponding antibiotic classes (reported in the rest of datasets)”. We have clarified that we are mapping “single” antibiotics. When combining the datasets, we average the resistance rates reported in each dataset for each combination of country, bacteria and antibiotic class; we have now clarified this in the main text to incorporate this comment’s suggestion.

***How much overlap was there between the datasets in country coverage? It would have been nice to have maps indicating country participation for the different datasets.***

 Thank you for this suggestion. We have decided not to include a new Figure, as we feel that this would lengthen the paper without directly relating to our research question. We now clearly state that there is geographical overlap in Table 1 by saying that ECDC data underlies the European data in ResistanceMap and GLASS, and we already stated the general geographical coverage of the datasets in that Table (Europe or Global).

***While you mention that you could only provide recommendations for bacteremia, pneumonia and cellulitis, in the app and table 3, meningitis and arthritis are still included?***

 The only recommendations we can provide for meningitis and arthritis come with the caveat that there is actually no data to inform bacterial susceptibility, and therefore rely on our assumption to consider lack of data equivalent to full susceptibility. This is stated in Table 3. As a consequence, we consider that we cannot give robust recommendations for these two syndromes. We have added a couple of sentences in the “Resulting recommendations on the appropriateness of empiric antibiotic therapies” section of the Results to highlight this.

***App***

***I think a line should be included explaining that the included AMR proportions are from hospital-associated infections. And a warning should be included that the data may not be representative of the national prevalence of AMR due to selective sampling (mainly tertiary care hospital data, selection of isolates with full AST data, i.e. the more resistant ones, etc.).***

 We have now added the following disclaimer line in the app: “Resistance prevalence reported here may not be representative of the national prevalence. This tool is for research use only. Do not use for clinical decision making.”

***The tool could have been even more useful if uploading of local resistance data was made easy. The possibility to export country-specific data could be helpful too. If this is easy to add, I would like to ask the authors to add this functionality, if it is a lot of work this would not be required.***

 We agree that it would be a nice feature to add to the app. Unfortunately, this would be complicated to add in practice, therefore we have not considered it at this stage, but something that we are looking into for future work.

***The cut-off for selecting first or second line antibiotics is binary which means that second line therapeutics could be advised even if resistance proportions only differ 1% point. Could it somehow be incorporated that only above a clinically relevant difference in resistance the advice would switch from first to second line antibiotics? As above, if this is easy to add, I would like to ask the authors to add this functionality, if it is a lot of work this would not be required. Then, I would like to see this remark added to the paper.***

 We agree that including subtleties around the levels of resistance and how this affects treatment choice would be great additions to this app, but we have decided not to include them in this iteration due to the level of work involved and the small likelihood that this happens frequently. As the user can alter the 15% cut-off this can be used to explore sensitivity and resistance levels. We have added a new section into the discussion about future iterations of the app and how this could be included.

**

***Discussion***

***In the limitations section, it should be discussed that these data may not be the most reliable source of AMR proportions to base empirical treatment on, especially since for certain countries the number of isolates included are very low and not very up-to-date (2017).***

 We now mention this in the fifth paragraph of the discussion.

***It should also be mentioned that the provided data focus on hospital-associated infections (and tertiary care centres) and do not apply to community-associated infections.***

 We now mention this in the fifth paragraph of the discussion.

***Finally, there should be a warning against the future use of ATLAS data without critical appraisal; there is very low “accuracy”, and too little information about the sampling scheme and its representativeness. On certain websites (*** ***https://www.amrindustryalliance.org/case-study/antimicrobial-testing-leadership-and-surveillance-atlas/*** ***) they claim that ”ATLAS is the only database of its kind capable of providing such a broad scope of reliable, readily available information in an easy to use platform” which is “crucial for clinicians and public health officials”, which could have important implications.***

 We now mention some cautionary advice regarding the ATLAS database in the second paragraph of the discussion and in the conclusion. Our primary purpose in this study wasn’t to appraise the ATLAS database, but to see how it could be used.

***Please also report about the future prospects of ATLAS data, and whether this database will remain available and/or will be updated with new data?***

 We have now added this information in the second paragraph of the discussion. To the best of our knowledge, the ATLAS database will remain available and be updated every 6 to 8 months.

# Peer review: Reviewer response for version 1

**DOI:** 10.21956/wellcomeopenres.16928.r36747

**Published:** 2019-10-23

**Authors:** Liam Shaw

**Affiliations:** 1 Nuffield Department of Clinical Medicine, University of Oxford, Oxford, UK

This is an important proof-of-concept study. The aim is to provide a "clinically-oriented presentation of resistance rates" from available AMR surveillance datasets. The main conclusion is that while this is technically possible for some syndromes, the datasets are not yet adequate.

 I enjoyed reading the manuscript and anticipate that it will be read with interest by other researchers interested in this general approach. Thanks to the authors for carrying out this work!

 Positive points:

- An impressive integration with existing datasets beyond the central dataset (ATLAS).
- A range of sources of data have been synthesised – not just resistance rates but also information on syndromes, guidelines, etc.
- Clear presentation of methodology and results.
- Online interactive app so readers can experiment themselves and get a feel for the approach.
- Reproducibility: I could successfully clone the github repository and run the app myself in under a minute.

I have selected "Approved with reservations" as I do have some suggestions for improvement, which I hope the authors will consider.

**Major points**

1. **Infection syndromes:**
  I think a strict definition of what is meant by “infection syndrome” should be given, as readers may not be that familiar with the term (I admit I wasn’t totally clear what was meant without looking at the examples). With the caveat that I am not a clinician, I think it is underplayed that the infection syndromes looked at are often not known to be due to bacteria. For example, upper respiratory tract infections – the majority are viral (the Further Methods state 90%). And for bacteraemia, patients can present only with fever, at which point a decision has to be made about treatment. It seems to me it would be less common to have a scenario where a patient has a known bacterial infection syndrome but nothing is known beyond that. This may be my own ignorance; I’d welcome any supporting evidence about how often this is the case in practice.
  I know the authors are aware of this problem, but I think it should be stated explicitly that the problem considered here is: “an ideal world where one was confident that an infection was bacterial but did not know the species or the resistance profile” (I think that’s a reasonable formulation?). The effect of including the proportion expected to be non-bacterial and therefore unaffected by antibiotics would be to reduce the likelihood of prescribing antibiotics. The high proportion of recommendations to treat with antibiotics beyond first-line antibiotics is driven by this exclusively bacterial assumption (and the resistance datasets used). This should be emphasised.
  To be clear, I’m not suggesting redoing all this work taking into account the proportion of patients presenting with symptoms X/Y/Z who have a bacterial vs. viral infection. The work here stands in its own right. However, the major problem in antibiotic prescribing is the decision to prescribe antibiotics *at all* rather than deciding whether to prescribe first/second/third-line antibiotics. I think this needs to be mentioned up front; at present it comes very late as the fourth limitation in the discussion. In practice this is what most readers will think of when thinking about antibiotic stewardship and how prescribing apps might help.
2. **Accuracy**
  I appreciate this is always put in quotes to emphasise the restricted sense of “accurate” that is meant, but I still think this is the wrong term. I suggest changing all uses of it and avoiding the word entirely. “Accurate” suggests that the ground truth or right answer can be derived from the ResistanceMap or ECDC dataset, and that because the ATLAS point estimate lies outside the 95% confidence interval from these datasets it is “inaccurate”, which I think is misleading. The right answer for the ‘true’ prevalence of resistance isn’t known in this circumstance, and in fact resistance prevalence is context-dependent on the sampling (community vs. hospital etc.). I’d suspect variation in sampling is the driver, as suggested, and also that the ATLAS dataset for some countries might include only a few sites. What is being defined is not accuracy, but agreement between datasets. So, “concordance” or “agreement” would be a better term.
  I would also be interested to see a plot using data presented in Supplementary Figure 1, but adding the agreement against the number of samples that went into producing the ATLAS point estimate. If the problem driving low agreement is not enough samples in the ATLAS dataset, then more samples would produce more agreement with ECDC. If the problem is differences in sampling (e.g. bias towards resistant organisms in ATLAS due to specific clinical setting) then more samples in ATLAS would produce *less* agreement with ECDC. It might not be as clear-cut as I’m assuming, but at the moment this isn’t clear. It seems like an important distinction to shed light on.
3. **Comparison with other datasets**
  I agree that ECDC data is merged into ResistanceMap and the resistance rates for those countries are therefore the same. Therefore, I’m a bit confused why a comparison is made of ATLAS vs. ECDC *and *ATLAS vs. ResistanceMap without apparently removing ECDC as far as I could tell? Fundamentally the ECDC data is inside ResistanceMap. It seems to me that the important comparisons would be ATLAS vs. ECDC and ATLAS vs. (ResistanceMap – ECDC). I think for this reason there’s currently double-counting going on in Supplementary Figure 1, 2, 3 which could be rectified. If I misunderstood and this has already been removed, apologies!

 

**Minor points**

 I have ordered these by section.

**Introduction**

- Para 1: First sentence, suggest replacing “being known” by “determined by tests” (as highlights that this can only be really known by some sort of test/procedure outside normal clinical assessment, and also that sometimes tests are inconclusive).
- Para 2: “infection syndrome” --> “infection syndromes”.
- Para 3: suggest providing example(s) of a bacterial infection syndrome where the aetiology varies by season.
- Para 4: think the usual phrase is “resource-limited” rather than “resource constrained” (I could be wrong)
- Para 5: “which have limited healthcare budgets and difficulties to access medicines” --> “which can have limited healthcare budgets and access to medicines”
- Para 6: “infection syndromes” need to be defined earlier. Suggest in Para 2.

 

**Methods**

- Para 2: “This dataset was made public in 2017” – I accept this was when the ATLAS website went live, but the dataset was made browsable, not downloadable. Worth explaining that the whole dataset as csv was actually released in 2018 to participants in Data Reuse Prize.
- Para 5, Surveillance data comparison: see major point about changing “accuracy” (and change elsewhere as well).
- (a) “Common infection syndromes”: I’m not sure if all these syndromes are identifiable with simple clinical examination and/or radiology, so just want to check this point i.e. what’s meant by “clinical examination”. As I mentioned, as far as I’m aware patients can present only with fever for bacteraemia, so I’m not sure how it can be easily identified. Also, the syndromes are assumed to be independent of each other i.e. patients present with only one. This is a limitation as in practice they can be linked e.g. bacterial meningitis develops from initial bacteraemia. Or e.g. NICE guidelines highlight link between hospital-acquired pneumonia and bacteraemia.  
- “NICE guideline” --> “NICE guidelines”. Also, the link to the NICE guidelines (reference 8) is wrong. It takes me to PHE’s 119-page document “Summary of antimicrobial prescribing guidance – managing common infections”. I think the correct link should be: https://www.nice.org.uk/Media/Default/About/what-we-do/NICE-guidance/antimicrobial%20guidance/summary-antimicrobial-prescribing-guidance.pdf Is this correct? Assuming these are the correct guidelines, I became slightly confused about how the syndromes were identified. For example, upper respiratory tract infection as defined in Table 2 doesn’t include “acute otitis media”, which is included in this syndrome in the NICE guidelines. The first-choice treatment for acute otitis media is amoxicillin, rather than penicillin V. I have not investigated all the other syndromes but I just want to check why this was excluded? Apologies if I missed this somewhere.
- On this point, I couldn’t find the guidelines for bacteraemia in that NICE link. “Bacteraemia” is the presence of bacteria in the blood; “sepsis” is probably a better term for the infection syndrome. I noticed that the app uses “sepsis”, but the main paper uses “bacteraemia”, suggest to make them consistent.  
- (d) “Contributing pathogen distribution: syndrome aetiology”: on “rapid literature search”, it would be good to know how this review worked in practice e.g. searching PubMed / Google Scholar / the wider web? One author assigned to a syndrome? One author doing all syndromes? How were results integrated? There’s no problem at all with it being rapid and informal, but a little more info on the process would be great.

 

**Results**

- Table 3: the summary table of the recommendations for therapy is based on the arbitrary 15% cut-off. This should be stated in the caption of the table. I think it would be good to present the actual distributions in a supplementary figure (i..e. the percentages for the index), but this isn’t essential. I would also state that only those syndromes with an average above 50% were included (which I infer from the associated text). Column stating “Average syndrome coverage” – presume “Mean syndrome coverage” is meant instead.
- (g) “Resulting recommendations on the appropriateness of empiric antibiotic therapies”
  Para 3: bacterial meningitis has an “average syndrome coverage” of 0.51 in Table 3, so why does a sentence here state it has lower than 50%? Also, suggest stating the syndrome coverages for these syndromes that are not listed in Table 3.
- Figures: in general, please check the resolution of the figures. For example, Figure 3 seems quite low-res to me. For Figure 1: the arrows on the left-hand side aren’t clear, I got a bit confused with the arrows pointing to other arrows. Suggest using a colour key for these arrows instead.
- Table 2: “Infectious syndrome” --> “Infection syndrome” (first column)

 

**Discussion**

- Para 3: “over-representation of antibiotic-resistant isolates in the ATLAS datasets” – see previous comment about a plot of ATLAS sample size against agreement with ECDC which might provide some more support for the hypothesis causing this.
- Para 5: “antimicrobial resistance” --> AMR

 

**Conclusion**

- “the WHO calls for” --> “the WHO’s call for”. Also provide a reference for this call for “evidence-based prescribing”. I wasn’t sure what was being referred to.

 

**Further methods**

- Section 1: no new MIC thresholds were included, which is acknowledged as a limitation. These would not in principle be difficult to include from EUCAST and could bump up the dataset. For example, *E. coli *and ceftaroline-avibactam: there are 13,203 isolates with a reported MIC. The EUCAST breakpoint is 0.5 mg/L. So using my version of the ATLAS dataset, 139/13,202 isolates (1.05%) are resistant. I accept compiling bug/drug combinations for breakpoints (and considering both EUCAST and CLSI) would be tedious, and so much data integration has already been done! I’d be happy to approve the paper without this extra work. But it would be good to have some idea of how much doing this would increase sample sizes, which could be done without external data integration.
- Section 3: This paragraph is quite dense and a brief overview in the main paper would be useful, because the index is a key concept. In a talk based on this work by Francesc Coll at a SEDRIC meeting on October 9 ^th^ 2019, I saw a great diagram, I think of the example given here. I would strongly suggest including this as a figure in the main paper, as it quickly conveys the idea of the index visually.

 

**Further results**

- Section 3: Upper respiratory tract infection has a reference (49) which is missing from the references at the end of this document.

Is the work clearly and accurately presented and does it cite the current literature?

Partly

If applicable, is the statistical analysis and its interpretation appropriate?

Yes

Are all the source data underlying the results available to ensure full reproducibility?

Yes

Is the study design appropriate and is the work technically sound?

Yes

Are the conclusions drawn adequately supported by the results?

Yes

Are sufficient details of methods and analysis provided to allow replication by others?

Yes

Reviewer Expertise:

My main research area is bacterial genetics, including antimicrobial resistance. I also have direct experience of analysing the ATLAS dataset used in this work.

I confirm that I have read this submission and believe that I have an appropriate level of expertise to confirm that it is of an acceptable scientific standard, however I have significant reservations, as outlined above.

# Response

**Published:** 2020-06-19

**Authors:** Quentin Leclerc

***This is an important proof-of-concept study. The aim is to provide a "clinically-oriented presentation of resistance rates" from available AMR surveillance datasets. The main conclusion is that while this is technically possible for some syndromes, the datasets are not yet adequate.***

******

***I enjoyed reading the manuscript and anticipate that it will be read with interest by other researchers interested in this general approach. Thanks to the authors for carrying out this work!***

******

Thank you for your comments and review, much appreciated. We have left your comments in bold and italics below, while our answers are written in plain text.

***Positive points:***

- ***An impressive integration with existing datasets beyond the central dataset (ATLAS). ***
- ***A range of sources of data have been synthesised – not just resistance rates but also information on syndromes, guidelines, etc. ***
- ***Clear presentation of methodology and results. ***
- ***Online interactive app so readers can experiment themselves and get a feel for the approach. ***
- ***Reproducibility: I could successfully clone the github repository and run the app myself in under a minute. ***

***I have selected "Approved with reservations" as I do have some suggestions for improvement, which I hope the authors will consider.***

******

***Major points***

1. ***Infection syndromes:*** I think a strict definition of what is meant by “infection syndrome” should be given, as readers may not be that familiar with the term (I admit I wasn’t totally clear what was meant without looking at the examples). With the caveat that I am not a clinician, I think it is underplayed that the infection syndromes looked at are often not known to be due to bacteria. For example, upper respiratory tract infections – the majority are viral (the Further Methods state 90%). And for bacteraemia, patients can present only with fever, at which point a decision has to be made about treatment. It seems to me it would be less common to have a scenario where a patient has a known bacterial infection syndrome but nothing is known beyond that. This may be my own ignorance; I’d welcome any supporting evidence about how often this is the case in practice.I know the authors are aware of this problem, but I think it should be stated explicitly that the problem considered here is: “an ideal world where one was confident that an infection was bacterial but did not know the species or the resistance profile” (I think that’s a reasonable formulation?). The effect of including the proportion expected to be non-bacterial and therefore unaffected by antibiotics would be to reduce the likelihood of prescribing antibiotics. The high proportion of recommendations to treat with antibiotics beyond first-line antibiotics is driven by this exclusively bacterial assumption (and the resistance datasets used). This should be emphasised.To be clear, I’m not suggesting redoing all this work taking into account the proportion of patients presenting with symptoms X/Y/Z who have a bacterial vs. viral infection. The work here stands in its own right. However, the major problem in antibiotic prescribing is the decision to prescribe antibiotics *at all* rather than deciding whether to prescribe first/second/third-line antibiotics. I think this needs to be mentioned up front; at present it comes very late as the fourth limitation in the discussion. In practice this is what most readers will think of when thinking about antibiotic stewardship and how prescribing apps might help.

Thank you for this comment. We have now added a clearer definition of “infection syndrome” in the second paragraph of the Introduction. In addition, we have added a statement in the last paragraph of the Introduction to clearly mention that we are assuming that we are in a scenario where an antibiotic has to be prescribed, and we therefore aim to answer the question of which antibiotic should be prescribed.

 

1. ***Accuracy*** ***I appreciate this is always put in quotes to emphasise the restricted sense of “accurate” that is meant, but I still think this is the wrong term. I suggest changing all uses of it and avoiding the word entirely. “Accurate” suggests that the ground truth or right answer can be derived from the ResistanceMap or ECDC dataset, and that because the ATLAS point estimate lies outside the 95% confidence interval from these datasets it is “inaccurate”, which I think is misleading. The right answer for the ‘true’ prevalence of resistance isn’t known in this circumstance, and in fact resistance prevalence is context-dependent on the sampling (community vs. hospital etc.). I’d suspect variation in sampling is the driver, as suggested, and also that the ATLAS dataset for some countries might include only a few sites. What is being defined is not accuracy, but agreement between datasets. So, “concordance” or “agreement” would be a better term.***

Thank you for this suggestion to improve clarity; we have now replaced “accuracy” by “agreement” throughout the manuscript.

***I would also be interested to see a plot using data presented in Supplementary Figure 1, but adding the agreement against the number of samples that went into producing the ATLAS point estimate. If the problem driving low agreement is not enough samples in the ATLAS dataset, then more samples would produce more agreement with ECDC. If the problem is differences in sampling (e.g. bias towards resistant organisms in ATLAS due to specific clinical setting) then more samples in ATLAS would produce less agreement with ECDC. It might not be as clear-cut as I’m assuming, but at the moment this isn’t clear. It seems like an important distinction to shed light on.***

Thank you for this suggestion. We have now divided Supplementary Figure 1 into two sections; A) is the old Supplementary Figure 1, and B) is a new plot showing agreement against number of ATLAS samples used to calculate that agreement. One sample is one isolate, for one bacteria, one antibiotic, and one country. This new plot suggests that more ATLAS samples produce more agreement with ECDC and ResistanceMap, however this does not seem clear-cut as the increase in agreement is arguably not significant past 30,000 samples. Therefore, this suggests that while increasing sample size can increase agreement, there is still a difference between the datasets, which could be due to differences in sampling. We now discuss this in the Extended Data – Further Results section, and have added a sentence on the difference in sampling between the datasets in the discussion section in the main text.

 

1. ***Comparison with other datasets*** ***I agree that ECDC data is merged into ResistanceMap and the resistance rates for those countries are therefore the same. Therefore, I’m a bit confused why a comparison is made of ATLAS vs. ECDC and ATLAS vs. ResistanceMap without apparently removing ECDC as far as I could tell? Fundamentally the ECDC data is inside ResistanceMap. It seems to me that the important comparisons would be ATLAS vs. ECDC and ATLAS vs. (ResistanceMap – ECDC). I think for this reason there’s currently double-counting going on in Supplementary Figure 1, 2, 3 which could be rectified. If I misunderstood and this has already been removed, apologies!***

Thank you for this suggestion. Our aim behind this comparison was to see how well the ATLAS dataset agreed with other AMR surveillance datasets, therefore we made all the comparisons against the full datasets (i.e. without removing the ECDC data from ResistanceMap). We believe this is the valid comparison to make as this will be the one that others are doing too: how do results from ATLAS analysis compare to results from ResistanceMap analysis rather than subsetting the latter database and comparing.

We agree that a more in-depth comparison between the AMR surveillance datasets would be interesting, however this would require a much larger analysis to be correctly examined. We therefore think that this is outside the scope of this paper as it would represent a different research question.

***Minor points***

***I have ordered these by section.***

******

***Introduction***

- ***Para 1: First sentence, suggest replacing “being known” by “determined by tests” (as highlights that this can only be really known by some sort of test/procedure outside normal clinical assessment, and also that sometimes tests are inconclusive).***

Thank you for this suggestion, we have made the recommended change to “determined by diagnostic tests”.

- ***Para 2: “infection syndrome” --> “infection syndromes”.***

Thank you for spotting this mistake, we have now corrected it.

- ***Para 3: suggest providing example(s) of a bacterial infection syndrome where the aetiology varies by season.***

We have now added the example of pneumonia as a bacterial infection syndrome where aetiology varies by season.

- ***Para 4: think the usual phrase is “resource-limited” rather than “resource constrained” (I could be wrong)***

We have made the recommended change to “resource-limited”.

- ***Para 5: “which have limited healthcare budgets and difficulties to access medicines” --> “which can have limited healthcare budgets and access to medicines”***

 We have rephrased this section as recommended.

- ***Para 6: “infection syndromes” need to be defined earlier. Suggest in Para 2.***

We have now added a definition of “infection syndromes” in paragraph 2 of the Introduction.

**

***Methods***

- ***Para 2: “This dataset was made public in 2017” – I accept this was when the ATLAS website went live, but the dataset was made browsable, not downloadable. Worth explaining that the whole dataset as csv was actually released in 2018 to participants in Data Reuse Prize.***

***​​​​​​​***Thank you for this clarification, we have added a sentence in paragraph 2 of the Methods to explain this.

- ***Para 5, Surveillance data comparison: see major point about changing “accuracy” (and change elsewhere as well).***

“accuracy” has now been replaced by “agreement” throughout the manuscript.

 

- ***(a) “Common infection syndromes”: I’m not sure if all these syndromes are identifiable with simple clinical examination and/or radiology, so just want to check this point i.e. what’s meant by “clinical examination”. As I mentioned, as far as I’m aware patients can present only with fever for bacteraemia, so I’m not sure how it can be easily identified. Also, the syndromes are assumed to be independent of each other i.e. patients present with only one. This is a limitation as in practice they can be linked e.g. bacterial meningitis develops from initial bacteraemia. Or e.g. NICE guidelines highlight link between hospital-acquired pneumonia and bacteraemia.  ***

Infection syndromes cover a wide range of possibilities and you are right that bacteraemia is notoriously difficult to pin down characteristic features for – fever is really the only reliably present element. Clinical examination essentially encompasses all the elements of medical examination. This would start with taking temperature and performing basic observations through to examining different parts of the body to look for localized evidence of infection.  Few of the infection syndromes have entirely reliable identification in practice. You are right that bacteraemia often co-exists with some of the other syndromes, but apart from that, overlap is relatively rare.

We have included further discussion of this in the discussion (paragraph 3).

- ***“NICE guideline” --> “NICE guidelines”. Also, the link to the NICE guidelines (reference 8) is wrong. It takes me to PHE’s 119-page document “Summary of antimicrobial prescribing guidance – managing common infections”. I think the correct link should be:*** ***https://www.nice.org.uk/Media/Default/About/what-we-do/NICE-guidance/antimicrobial%20guidance/summary-antimicrobial-prescribing-guidance.pdf*** ***Is this correct? Assuming these are the correct guidelines, I became slightly confused about how the syndromes were identified. For example, upper respiratory tract infection as defined in Table 2 doesn’t include “acute otitis media”, which is included in this syndrome in the NICE guidelines. The first-choice treatment for acute otitis media is amoxicillin, rather than penicillin V. I have not investigated all the other syndromes but I just want to check why this was excluded? Apologies if I missed this somewhere.***

Thank you for the corrected link. For “acute otitis media” (AOM), even though this infection is frequently caused by bacteria, there are typically no bacterial samples sent for patients. This is because AOM occurs on the inside of eardrum, hence it is hard to access unless the eardrum is perforated. We confined our work to infection syndromes where there were easily corresponding bacterial isolates. To make recommendations about AOM, one would normally draw inferences from bacteria isolates from other body sites, which we have avoided doing here.  

Broadly, we agree with the reviewer that this is a limitation of our analysis. We aimed to provide a tool that could be used at the point of prescribing antibiotics to treat an “infection syndrome”. We therefore make no clear statements about whether these are independent or upon what level of evidence the decision is made. We have added this as a limitation into our discussion section (paragraph 3).

- ***On this point, I couldn’t find the guidelines for bacteraemia in that NICE link. “Bacteraemia” is the presence of bacteria in the blood; “sepsis” is probably a better term for the infection syndrome. I noticed that the app uses “sepsis”, but the main paper uses “bacteraemia”, suggest to make them consistent.  ***

You are correct that there is no mention of bacteraemia in the NICE guidelines – we felt that we should add this as a recommendation category. The terms sepsis and bacteraemia are often used interchangeably, though they refer to different phenomena. For consistency, we will use the term “bloodstream infection” as the standard description for both the app and the paper. 

- ***(d) “Contributing pathogen distribution: syndrome aetiology”: on “rapid literature search”, it would be good to know how this review worked in practice e.g. searching PubMed / Google Scholar / the wider web? One author assigned to a syndrome? One author doing all syndromes? How were results integrated? There’s no problem at all with it being rapid and informal, but a little more info on the process would be great.***

Thank you for this suggestion. We have now added this information in the main text.

**

***Results***

- ***Table 3: the summary table of the recommendations for therapy is based on the arbitrary 15% cut-off. This should be stated in the caption of the table. I think it would be good to present the actual distributions in a supplementary figure (i..e. the percentages for the index), but this isn’t essential. I would also state that only those syndromes with an average above 50% were included (which I infer from the associated text). Column stating “Average syndrome coverage” – presume “Mean syndrome coverage” is meant instead.***

We now mention the 15% cut-off in the caption, and have corrected the column to indicate “Mean syndrome coverage”. In the Extended Data (Further Methods) section we acknowledge that the 15% cut-off is rather conservative compared to the 25% resistance rate recommended by European guidelines for managing hospital-acquired and ventilator-acquired pneumonia ( *1*[QL1] ). Hence why we allow the user to vary this in the app.

- ***(g) “Resulting recommendations on the appropriateness of empiric antibiotic therapies”Para 3: bacterial meningitis has an “average syndrome coverage” of 0.51 in Table 3, so why does a sentence here state it has lower than 50%? Also, suggest stating the syndrome coverages for these syndromes that are not listed in Table 3.***

***​​​​​​​***Thank you for spotting this mistake, bacterial meningitis should not have been in this sentence. We have now corrected that, and give the syndrome coverage values in the text for the two syndromes with a coverage of less than 50%.

- ***Figures: in general, please check the resolution of the figures. For example, Figure 3 seems quite low-res to me. For Figure 1: the arrows on the left-hand side aren’t clear, I got a bit confused with the arrows pointing to other arrows. Suggest using a colour key for these arrows instead.***

Thank you for pointing this out, we have now edited all Figures to be of higher resolution, and have added two types of arrows in Figure 1 to hopefully make this figure clearer.

- ***Table 2: “Infectious syndrome” --> “Infection syndrome” (first column)***

We have now corrected this.

**

***Discussion***

- ***Para 3: “over-representation of antibiotic-resistant isolates in the ATLAS datasets” – see previous comment about a plot of ATLAS sample size against agreement with ECDC which might provide some more support for the hypothesis causing this.***

***​​​​​​​***Thanks again for this comment – we have now added an extra sentence here to highlight the differences in sampling between the datasets.

- ***Para 5: “antimicrobial resistance” --> AMR***

We have corrected this to the recommended abbreviation.

**

***Conclusion***

- ***“the WHO calls for” --> “the WHO’s call for”. Also provide a reference for this call for “evidence-based prescribing”. I wasn’t sure what was being referred to.***

Thank you for highlighting this, we have corrected the sentence and added a reference for this call.

**

***Further methods***

- ***Section 1: no new MIC thresholds were included, which is acknowledged as a limitation. These would not in principle be difficult to include from EUCAST and could bump up the dataset. For example, E. coli and ceftaroline-avibactam: there are 13,203 isolates with a reported MIC. The EUCAST breakpoint is 0.5 mg/L. So using my version of the ATLAS dataset, 139/13,202 isolates (1.05%) are resistant. I accept compiling bug/drug combinations for breakpoints (and considering both EUCAST and CLSI) would be tedious, and so much data integration has already been done! I’d be happy to approve the paper without this extra work. But it would be good to have some idea of how much doing this would increase sample sizes, which could be done without external data integration.***

Thank you for raising this interesting point. We did not consider this further data integration when completing this work, however you are undeniably correct when saying that it would increase the number of useful data points in the ATLAS dataset. The issue would come with deciding which guideline to follow and to split our analysis further by EUCAST and CLSI for example would indeed by a large amount of extra work. We did do some of this work by using existing breakpoints in the data for isolates without a classification but an MIC value.

We have now added a few sentences at the end of this section to clarify that we did not do this, but that it could potentially be applied to increase the value of the dataset. We also would note that many of the antibiotics where the additional data would be added (eg ceftaroline-avibactam, colistin etc) are very “high-level” antibiotics that would not feature in empirical guidelines. 

- ***Section 3: This paragraph is quite dense and a brief overview in the main paper would be useful, because the index is a key concept. In a talk based on this work by Francesc Coll at a SEDRIC meeting on October 9 ^th^ 2019, I saw a great diagram, I think of the example given here. I would strongly suggest including this as a figure in the main paper, as it quickly conveys the idea of the index visually.***

We thank you for this suggestion and have now included a further figure (Figure 3) based on        the slide presented.  

***Further results***

- ***Section 3: Upper respiratory tract infection has a reference (49) which is missing from the references at the end of this document.***

Thank you for spotting this mistake. We have corrected this to indicate the correct reference number (17), and have added the reference in the bibliography.

 [QL1]A. Torres *et al.*, International ERS/ESICM/ESCMID/ALAT guidelines for the management of hospital-acquired pneumonia and ventilator-associated pneumonia. *European Respiratory Journal*. **50**, 1700582 (2017).
